# Backtranslation of human RNA biosignatures of tuberculosis disease risk into the preclinical pipeline is condition dependent

**DOI:** 10.1128/msphere.00864-24

**Published:** 2024-12-09

**Authors:** Hannah Painter, Sasha E. Larsen, Brittany D. Williams, Hazem F. M. Abdelaal, Susan L. Baldwin, Helen A. Fletcher, Andrew Fiore-Gartland, Rhea N. Coler

**Affiliations:** 1 Department of Infection Biology, London School of Hygiene and Tropical Medicine, London, United Kingdom; 2 Center for Global Infectious Disease Research, Seattle Children’s Research Institute, Seattle, Washington, USA; 3 Department of Global Health, University of Washington, Seattle, Washington, USA; 4 Biostatistics, Bioinformatics and Epidemiology Program, Fred Hutch Cancer Center, Seattle, Washington, USA; 5 Department of Pediatrics, University of Washington School of Medicine, Seattle, Washington, USA; Washington University in St. Louis School of Medicine, St. Louis, Missouri, USA

**Keywords:** tuberculosis, tuberculosis vaccines, RNA risk signature, correlate of risk, preclinical drug studies

## Abstract

It is unclear whether human progression to active tuberculosis disease (TB) risk signatures are viable endpoint criteria for evaluations of treatments in development. TB is the deadliest infectious disease globally and more efficacious vaccines are needed to reduce this mortality. However, the immune correlates of protection for either preventing infection with *Mycobacterium tuberculosis* or preventing TB disease have yet to be completely defined, making the advancement of candidate vaccines through the pipeline slow, costly, and fraught with risk. Human-derived correlate of risk (COR) gene signatures, which identify an individual’s risk of progressing to active TB disease, provide an opportunity for evaluating new therapies for TB with clear and defined endpoints. Though prospective clinical trials with longitudinal sampling are prohibitively expensive, the characterization of COR gene signatures is practical with preclinical models. Using a 3Rs (replacement, reduction, and refinement) approach we reanalyzed heterogeneous publicly available transcriptional data sets to determine whether a specific set of COR signatures are viable endpoints in the preclinical pipeline. We selected RISK6, Sweeney3, and BATF2 human-derived blood-based RNA biosignatures because they require relatively few genes and have been carefully evaluated across several clinical cohorts. These data suggest that in certain experimental designs and in several tissue types, human COR signatures correlate with disease progression as measured by the bacterial burden in the preclinical TB model pipeline. We observed the best performance when the model most closely reflected human infection or disease conditions. Human-derived COR signatures offer an opportunity for high-throughput preclinical endpoint criteria of vaccine and drug therapy evaluations.

**IMPORTANCE:**

Understanding the strengths or limitations of back-translating human-derived correlate of risk (COR) RNA signatures into the preclinical pipeline may help streamline down-selection of therapeutic vaccine and drug candidates and better align preclinical models with proposed clinical trial efficacy endpoints.

## INTRODUCTION

Tuberculosis disease (TB), caused by the bacterial pathogen *Mycobacterium tuberculosis* (M.tb), was the leading infectious killer globally for 4 years (1) predating the severe acute respiratory syndrome coronavirus 2 pandemic and was again the widespread frontrunner from 2022 (2). Approximately 1.3 million deaths, including over 150,000 people living with HIV, in 2023 worldwide were attributed to TB (3). The World Health Organization estimates that coronavirus disease 2019-related disruptions in care could result in an additional half million deaths from TB (4). The burden of TB continues to disproportionately affect low- and middle-income countries (5). While the classic infection risks for M.tb are fairly well-known and studied (6–10), the majority of individuals who harbor M.tb do not advance to active TB disease (11–15). Less well-known are the factors that influence an individual’s progression through the spectrum of TB disease (14, 16, 17), or what determines their risk of advancing to an active, transmissible state. Indeed, the current methods to identify M.tb infection, the tuberculin skin test, and interferon-γ release assay, have poor specificity for TB disease in endemic populations, including for those individuals living with HIV (18, 19). The TB field needs better triage criteria that can allow targeted identification of active and incipient subclinical TB disease.

Highly specific predictive correlate of risk (COR) biomarkers or diagnostics could enable risk-stratified preventive interventions. These tools may avoid unnecessary treatment of people who would likely remain healthy, while also interrupting incipient TB disease in infected adults, leading to a reduction in morbidity and transmission. Studies are ongoing to evaluate several clinical applications (20–23). Human peripheral blood-based biosignatures have been able to selectively identify TB disease from other infections, distinguish latent TB infection (LTBI), and predict progression to active TB disease (24–29). Correlative biomarkers for TB-related disease states are well reviewed here (30–32). Iterative large-scale human clinical trials to examine the performance of multiple host biomarkers would be cost-prohibitive, however, assessing human-derived RNA risk signatures of progression to active disease in the preclinical pipeline may facilitate the selection of efficacy endpoints used to screen products and accelerate preclinical development.

Understanding the strengths or limitations of back-translating human-derived COR RNA signatures into the preclinical pipeline may help streamline down-selection of therapeutic vaccine and drug candidates and better align preclinical models with proposed clinical trial efficacy endpoints. However, to date, few published studies have evaluated the appropriateness of leveraging human-derived RNA COR gene signatures, including RISK6, Sweeney3, or BATF2, in preclinical models (33). Given the abundance of host-derived gene expression data published, in this work, we identified existing public transcriptomic data sets across the spectrum of preclinical TB disease models (mouse, guinea pig [GP], rabbit, and non-human primates [NHP]) and determined expression of select COR gene signatures and risk score correlation with disease endpoints. Our primary objective was to determine if human-derived RNA gene signatures predicting progression to active TB disease could be back-translated into animal models. Our secondary objective was to identify whether certain experimental conditions, such as infection dose, were limitations for RNA COR signature use. We hypothesized that along with bacterial burden, pulmonary inflammation-induced damage may correlate with increasing COR gene signatures across preclinical models. Furthermore, we aimed to identify models of vaccine and drug interventions that lower COR gene expression.

## RESULTS

A considerable number of validated human COR signatures have been published to date. These signatures vary sizably from hundreds to a single gene target (32, 34). The published preclinical data sets identified in our searches were diverse with respect to both the model species and the method (microarray vs RNAseq) utilized to perform transcriptional analysis. To facilitate the inclusion of the largest possible number of data sets we selected three parsimonious (1–6 genes) COR biosignatures, RISK6 (27), Sweeney3 (35), and BATF2 (36), for use in these analyses (Table 1). In published human cohort studies, a higher number of genes was not found to correlate with better performance in a head-to-head comparison of 16 different biosignatures (37). The three biosignatures chosen for this work were included in two critical meta-analyses, which used independent validation cohorts to demonstrate that sensitivity and specificity remain high, particularly when in close proximity to TB disease status (32, 34). Interestingly, many genes contributing to each signature score are present across several gene signatures developed in human clinical studies (see Table S2 [gene matrix] in reference 34) and have direct immune regulatory functions (Table S1). Furthermore, Sweeney3 and RISK6 are under development as point-of-care tests and have been shown to correlate with pathology scores in humans (27, 38, 39). Pathology scores can be readily assessed in the majority of preclinical animal models of TB, and a number of the gene expression data sets reanalyzed in this study included pathology endpoints.

**TABLE 1:** Description of down-selected human TB transcriptional risk signatures

Name	Gene composition	Score equation	Discovery cohort	Current applications
RISK6 (27)	GBP2, FCGR1B,^*a*^ SERPING1, TUBGCP6, TRMT2A, SDR39U1	Geometric mean(TUBGCP6, TRMT2A, SDR39U1) – geometric mean(GBP2, FCGR1B, SERPING1)	HIV-negative South African adolescents (Adolescent Cohort Study [29]): 46 “progressors” matched to “non-progressors” (AUC 87.6%, 95% CI 82.8–92.4)	TB disease risk, diagnosis, and treatment response
Sweeney3 (35)	GBP5, DUSP3, KLF2	Mean(GBP5 + DUSP3) – KLF2	HIV-positive and -negative adults, various sites from three published data sets (GSE19491 [24], GSE37250 [26], and GSE42834 [40]): 236 LTBI, 491 other diseases and 296 active TB (active TB vs healthy controls: AUC 0.96, 95% CI 0·94–0.98, and 1.0, 95% CI 1–1; latent tuberculosis: AUC 0.93, 95% CI 0.91–0.95 and 0.93, 95% CI 0.91–0.94; other diseases: mean AUC 0.88, range 0.84–0.92)	TB disease risk, diagnosis, disease severity, and treatment response
BATF2 (36)	BATF2	BATF2	HIV-negative UK adults (AdjuVIT Cohort) (46 active TB cases, and 31 non-TB controls: ROC 0.99)	TB disease risk and diagnosis; differentiation between pulmonary and extra-pulmonary TB

^*a*^ In all murine data sets, the mouse ortholog Fcgr1 was used; NHP Ahmed, FCGR1; Gideon, FCGR1B; Hansen, FCGR1A. Otherwise, gene homologs were used for each species.

This reanalysis of published data demonstrates that genes used in select human COR signatures are also able to predict progression to active TB disease in some preclinical models of M.tb infection in the context of the spectrum of TB disease (naïve, latent, and active), as well as identifying drug treatment success. These signatures may be suitable for use as surrogate vaccine intervention endpoints with appropriate kinetic considerations.

### Primary data discovery and inclusion-exclusion criteria

Searches for primary data for inclusion in this reanalysis work were executed through the Gene Expression Omnibus (GEO) repository as well as PubMed. We chose to include *in vivo* preclinical models of TB experimental infection including mice, GPs, rabbits, and NHPs to cover a spectrum of disease outcomes and intervention strategies. GEO entries were collated with the following search terms “mus tuberculosis AND Mus musculus” (69 total hits), “macaca tuberculosis” (4 total hits), “oryctolagus tuberculosis” (7 total hits), and “cavia tuberculosis” (1 hit). Since some data is presented in supplemental figures and not directly stored in GEO, we executed tandem searches in PubMed using combinations of the following terms: tuberculosis, M.tb, biomarkers, RNA, risk scores, preclinical models, mouse, NHP, guinea pig, rabbit, challenge, microarray, sequencing, host gene expression, vaccine, or drug treatment. Four non-redundant data sets were identified on PubMed that were not present in results from GEO searches. The aim of this work was to evaluate whether three selected biosignatures can be readily applied across the preclinical pipeline. We therefore omitted data involving cell lines or sorted primary cells, or single-cell RNA-seq data, since these source tissues add complexity to analysis and are more mechanistic (Table 2). Inclusion criteria for gene expression data for the reanalysis involved were data derived from non-review sources; data generated from tissue within a preclinical *in vivo* experimental infection study; study group size within the data set must be ≥2; and data set contains at least one of the three selected biosignatures (all genes within each biosignature must be present) (Table 2). While data sets with *n* = 2 were collected, they were used for observational trends only and not assessed statistically.

**TABLE 2:** Selection of data for analysis

Inclusion criteria	Exclusion criteria
Contains primary data, non-review based	Single-cell RNA-seq
Contains complete gene set in at least one biosignature	Cell lines
Based on tissue derived from *in vivo* preclinical models of TB experimental infection	Sorted primary cell subsets
Group size *n* ≥ 2	

Through GEO and PubMed we identified a total of 30 sources of primary gene expression data meeting our inclusion and exclusion criteria which were further reanalyzed for this work (Tables 3 to 5). Data sets identified but excluded can be found in Table S2 with the rationale for exclusion. These sources provided 27 *Mus musculus* (mouse) and 3 *Macaca* (NHP) data sets. Data selected for reanalysis represents a spectrum of models with different experimental infection doses (Table 3), kinetics with respect to time since infection (Table 4), tissues of interest, as well as vaccine, and chemotherapeutic interventions (Table 5). Throughout we consistently use the primary authors’ definition of infection dose as “high” or “low” and state CFU range as reported in Materials and Methods.

**TABLE 3:** Data sets involving different infection doses of M.tb^*a*^

GEO (reference)	First author; year	Sample	Species	Sex	Infection dose	M.tb strain(s)
GSE158807 (33)	Plumlee; 2020	WB	*Mus musculus*; C57Bl/6	F	Ultra-low: 1–3 CFU; conventional: 50–100 CFU	H37Rv
n/a (41)	Ahmed; 2020	Lung	*Macaca mulatta*	M and F	Low: 10 CFU; high: 100 CFU	CDC1551
GSE179417 (42)	Koyuncu; 2021	Lung	*Mus musculus*; D.O.	F	25–100 CFU	Erdman
n/a (41)	Ahmed; 2020	Lung	*Mus musculus*; D.O.	M and F	100 CFU	HN878
GSE137093 (43)	Moreira-Teixeira; 2019	Lung	*Mus musculus*; C57Bl/6 and C3HeB/FeJ	F	At day 3: low: 100–450 CFU; high: 700–900	H37Rv and HN878
GSE137092 (43 )	Moreira-Teixeira; 2019	WB	*Mus musculus*; C57Bl/6 and C3HeB/FeJ	F	At day 3: low: 100–450 CFU; high: 700–900	H37Rv and HN878

^*a*^ D.O., diversity outbred mice; WB, whole blood sample. M.tb infection is via aerosol unless otherwise noted.

**TABLE 4:** Data sets involving kinetic time points post-infection^*a*^

GEO (reference)	First author; year	Sample	Species	Sex	Infection dose	M.tb strain	Sampling days post-infection
GSE124688 (44)	Ault; 2019	WB	*Mus musculus*; C57Bl/6	F	50–100 CFU	Erdman	30, 60, 90, 120, and 150
GSE21149 (45)	Aranday-Cortes; 2011	Lung, spleen	*Mus musculus*; BALB/c	F	600 CFU	*Mycobacterium bovis* (i.n.)	0, 3, and 14
GSE140944 (43)	Moreira-Teixeira; 2019	Lung	*Mus musculus*; BALB/c	F	100–1,000 CFU	H37Rv	0, 14, 21, 28, 57, and 129
GSE140943 (43)	Moreira-Teixeira; 2019	Blood	*Mus musculus*; BALB/c	F	100–1,000 CFU	H37Rv	0, 14, 21, 28, 56, and 138
GSE89389 (46)	Domaszewska; 2017	WB	*Mus musculus*; C57Bl/6 and 129S2	M and F	“Low dose”	H37Rv	0, 1, 7, 14, and 21
GSE64045 (47)	Mehra; 2014	Lung	*Mus musculus*; C3HeB/FeJ	M	~100 CFU	H37Rv	28, 56, and 112
GSE23014 (48)	Kang; 2010	Lung	*Mus musculus*; C57Bl/6	M and F	100 CFU	H37Rv	0, 12, 15, and 21
GSE84152 (49)	Gideon; 2016	WB	*Macaca fascicularis*	M and F	25 CFU	Erdman (instill.)	0, 1, 3, 7, 10, 20, 30, 42, 56, 90, 120, 150, and 180
GSE169541 (50)	Cerezo-Cortés; 2021	Lung	*Mus musculus*; BALB/c	M	250 CFU	Beijing-like (instill.)	3, 14, 28, and 60
GSE168486 (51)	Naqvi; 2021	Lung	*Mus musculus*; C57Bl/6 and MGL-1^−/−^	M and F	100 CFU	H37Rv (i.n.)	14 and 56

^*a*^ M.tb infection is via aerosol unless otherwise noted; i.n., intranasal infection; instill, intratracheal or intrabronchial instillation; WB, whole blood.

**TABLE 5:** Data sets involving anti-M.tb interventions^*a*^

GEO (reference)	First author; year	Sample	Species	Sex	Infection dose	M.tb strain	Intervention
GSE64167 (52)	Wilk; 2013	Lung	*Mus musculus*; A/Sn and I/St	M	5 × 10^6^ CFU	H37Rv (i.v.)	Genetic
GSE166114 (53)	Ji; 2021	Lung	*Mus musculus*; C57Bl/6 and Sp110 and Sp140	M and F	20–50 CFU	Erdman	Genetic
GSE165871 (54)	Bohrer; 2021	Lung	*Mus musculus*; C57Bl/6 and ΔdblGata	M and F	Low: 100–300 High: 1,000–1,500 CFU	H37Rv	Genetic
GSE141207 (55)	Moreira-Teixeira; 2019	WB and lung	*Mus musculus*; C57Bl/6	F	200 CFU	HN878	α-GM-CSF
GSE55183 (56)	Dutta; 2013	Lung	*Mus musculus*; C3HeB/FeJ	F	“OD_600_ approx. 1.0”	H37Rv	α-TNF
GSE127263 (57)	Matarese; 2019	Spleen	*Mus musculus*; DBA/2	F	10^5^ CFU	H37Rv (i.v.)	Caloric restriction
GSE57275 (58)	Singal; 2014	Lung	*Mus musculus*; C57Bl/6	F	10^3^ CFU	H37Rv	Metformin
GSE179437 (59)	Gandotra; 2021	Lung	*Mus musculus*; C3HeB/FeJ	M and F	Low: 100 High: 500 CFU	Erdman	T863
GSE99625 (60)	Dutta; 2017	Lung	*Mus musculus*; BALB/c	F	5 × 10^3^ CFU	H37Rv	RHZE
GSE83188 (61)	Subbian; 2016	Lung	*Mus musculus*; B6D2F1	F	100–150 CFU	CDC1551	CC 11050
GSE48027 (62)	Manca; 2013	Lung	*Mus musculus*; B6D2F1	F	2.5 log_10_	CDC1551	PZA
GSE44825 (63)	Rodrigues; 2013	Lung	*Mus musculus*; BALB/c	F	10^5^ CFU	H37Rv (instill.)	RH
GSE97835 (64)	Park; 2017	Lung	*Mus musculus*; C57Bl/6	n/a	200–300 CFU	Erdman	HZE
GSE102440 (65)	Hansen; 2017	WB	*Macaca mulatta*	M	10–25 CFU	Erdman (instill.)	Vaccination

^*a*^ M.tb infection is via aerosol unless otherwise noted. E, ethambutol; GM-CSF, granulocyte-macrophage colony-stimulating factor; H, isoniazid; i.v., intravenous infection; instill, intratracheal or intrabronchial instillation; R, rifampin; TNF, tumor necrosis factor; WB, whole blood; Z/PZA, pyrazinamide.

### Dose

Refinement of preclinical aerosol infection models has allowed TB researchers to consistently tailor the infection doses of M.tb from as few as one bacillus to several thousand (33, 66). These developments better represent the current understanding of natural human exposure to M.tb where few or one bacilli can establish infection. Since host COR signatures have been demonstrated to aid prediction of progression to active disease, often corresponding with loss of control of M.tb in granulomas and expansion of the bacillary load, we hypothesized that infection dose may positively correlate with COR signature expression in these data sets. This reanalysis encompassed six data sets meeting our inclusion/exclusion criteria which included NHP (*Macaca mulatta*) and mouse (diversity outbred [D.O.], C57BL/6, and C3HeB/FeJ) samples derived from lungs and whole blood (WB), across several M.tb strains including M.tb HN878, H37Rv, CDC1551, and Erdman (Table 3).

#### Impact of M.tb infection dose on pulmonary bacterial burden and signature score expression

**Fig 1:**
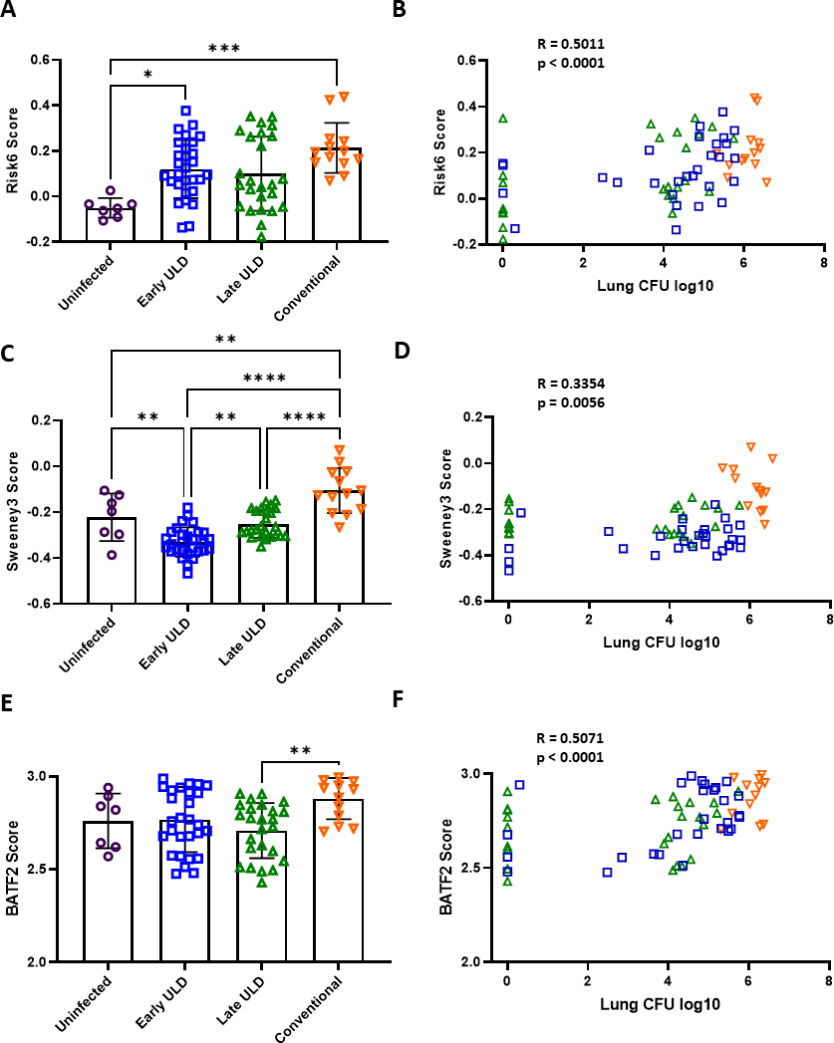
Risk signature scores by infection dose in a mouse model. In Plumlee et al. (GSE158807) C57BL/6 mice were infected with a CD/LDA (50–100 CFU) or ULDA (1–3 CFU) of M.tb H37Rv and host RNA was collected from WB at 24-days post-infection. Samples were analyzed using Agilent-074809 SurePrint G3 Mouse GE v2 8×60K Microarray and compared with naïve (open purple circles) at early (ULDA only, open blue squares) and late time points (CD: open orange upside-down triangle and ULDA: open green triangle). (**A**) Risk6, (**C**) Sweeney3, and (**E**) BATF2 scores were calculated for each mouse and cohorts were compared using one-way analysis of variance (ANOVA) with Tukey’s multiple comparison test correction. Significant comparisons are denoted by asterisks in the figure where * = *P* ≤ 0.05, ** = *P* ≤ 0.01, *** = *P* ≤ 0.001, **** = *P* ≤ 0.0001. Log_10_ lung CFU values were matched for each animal to (**B**) Risk6, (**D**) Sweeney3, and (**F**) BATF2 scores for correlation using a Spearman rank-based correlation test. *R* and *P* values are shown for each test in the figure.

The Plumlee et al. (GSE158807) data set contained the lowest infection dose and a higher, conventional dose (CD) (33). We compared risk signature scores in WB isolated from naïve mice, mice infected with a low-dose aerosol (LDA; 50–100 CFU, also termed CD by the authors), and ultra-low dose aerosol (ULDA; 1–3 CFU) infected mice. We aimed to determine whether mouse-level variability in signature expression correlated with dose at the time of infection and/or resulting CFU at designated endpoints. In general, each score was able to differentiate CD infection from either naïve mice or ULDA mice (Fig. 1). Both CD and early ULDA Risk6 scores were higher than naïve, with a general increase in score between ULDA and CD although this was not significant (Fig. 1A). Sweeney3 scores between cohorts were able to significantly differentiate between each group, again where CD scores were higher than ULDA (Fig. 1B). Interestingly BATF2 scores were similar between all groups tested and the only difference observed was between late-isolated ULDA and CD (Fig. 1C). In addition, this study had corresponding lung CFU metadata that could be compared with scores between groups. When comparing lung CFU values with each animal score we observe that Risk6 (Fig. 1D), Sweeney3 (Fig. 1E), and BATF2 (Fig. 1F) scores each correlate with pulmonary bacterial burden. It is difficult to disentangle whether these relationships between signature expression and outcome CFU are singular or also involve early innate responses to infection not evaluated here. Overall Risk6 variability correlated strongest with outcome CFU (*R* = 0.5011, *P* < 0.0001) independent of the infection treatment group, observed in Fig. 1B where group colors overlap. Similarly, BATF2 correlates with the outcome even though it is equal among the challenge doses, suggesting it has more to do with the outcome than the initial challenge. Whereas, Sweeney3 scores seem to separate more strongly by infection treatment group (Fig. 1D).

#### Signature score expression in controlled or progressive TB disease models

**Fig 2:**
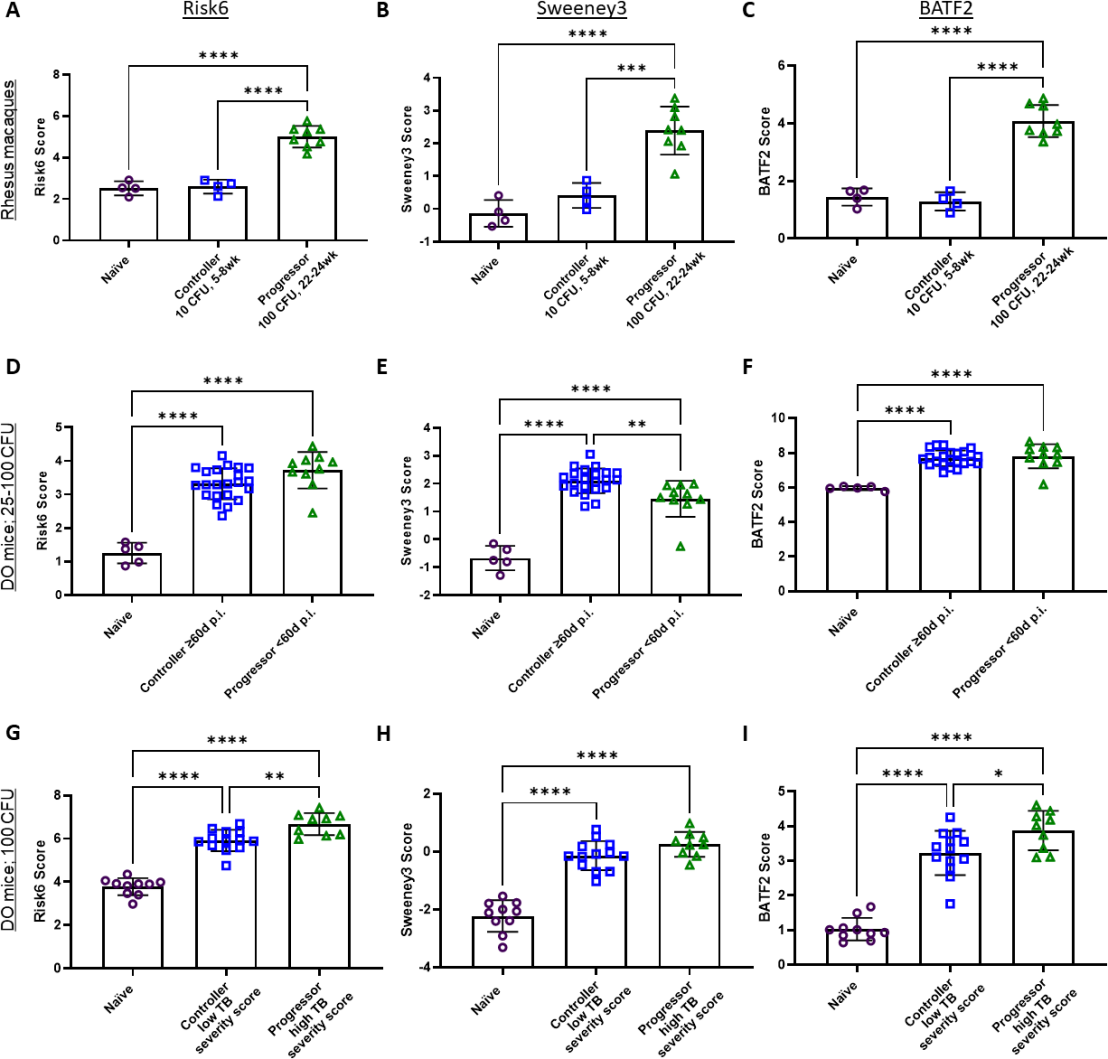
Risk signature scores assigned to naïve (open purple circles), controller (open blue squares), or progressor (open green triangles) NHP or DO mouse lung samples. (**A–C**) From Ahmed et al. (41), Indian rhesus macaques were infected with either 10 CFU and lung tissue obtained at 5–8 weeks post-infection (controllers), or 100 CFU M.tb CDC1551 and tissue collected 22–24 weeks post-infection (progressors) for RNA-seq. (**D–F**) In Koyuncu et al. (GSE179417), DO mice were infected with 25–100 CFU M.tb Erdman, and lung RNA was analyzed by Affymetrix Mouse Gene 2.0 ST Array (GSE179417). Progressors met endpoint morbidity criteria and controllers persisted out to 60 days post-infection. (**G–I**) In Ahmed et al. (41), DO mice were infected with 100 CFU M.tb HN878, and were assigned progressors or controllers based on “TB severity score” combining lung bacteria burden and inflammation. (**A, D, G**) Risk6, (**B, E, H**) Sweeney3, and (**C, F, I**) BATF2 scores were calculated for each animal, and cohorts were compared using one-way ANOVA with Tukey’s multiple comparison test correction. Significant comparisons are denoted by asterisks in the figure where * = *P* ≤ 0.05, ** = *P* ≤ 0.01, *** = *P* ≤ 0.001, **** = *P* ≤ 0.0001.

Three studies categorized cohorts as “controllers” or “progressors” after infection with M.tb and are presented here. In Ahmed et al. (41), Indian rhesus macaques were infected with either 10 CFU M.tb CDC1551, and lung tissue was collected at 5–8 weeks post-infection (controllers), or 100 CFU M.tb CDC1551 and samples collected 22–24 weeks post-infection (progressors). Gene expression analysis was performed by RNA-seq. Koyuncu et al. (GSE179417) infected D.O. mice with 25–100 CFU M.tb Erdman and lung RNA was analyzed by Affymetrix Mouse Gene 2.0 ST Array (42) (GSE179417). DO mouse progressors in the study met endpoint morbidity criteria (most near 20 days post-infection) whereas controllers remained above morbidity thresholds at 60 days post-infection. In the third study DO mice were infected with 100 CFU M.tb HN878, and assigned as progressors or controllers based on a severity score including log_10_ CFU, area of lung inflammation, and *a priori* threshold scores to segregate groups (41). In all three datasets, Risk6, Sweeney3, and BAFT2 scores were able to significantly discern naïve animals from progressor groups (Fig. 2). Ability to discriminate between controllers and naïve animals, as well as between controllers and progressors was less robust and varied between model species and score. Scores in controllers in the NHP data set were significantly reduced compared with the progressors, and undiscernible from naïve animals (Fig. 2A through C). All signature scores were found to significantly correlate with lung bacterial burden and percent lung inflammation endpoints in the NHP study (Fig. S1). By contrast, in the DO mouse data sets, scores in the controller groups were more comparable to progressor than the naïve animals, and discrimination between controller and progressor groups by the three signatures was not consistent (Fig. 2D through I). Scores in the Ahmed DO mouse data sets were also found to strongly correlate with lung bacterial burden (Fig. S2A, C, and E). The correlation between % lung inflammation and scores was weaker (Fig. S2B, D, and F).

In addition to the possibility that variations in the performance of the signatures may be driven by differences in species (NHP vs DO mouse), we hypothesize that these differences may be related to the method by which progressor and controller infections were established. For example, in the NHP Ahmed data set, where progressor and controller groups were established by dose and duration of infection, lung CFU in controllers at the study end ranged between no bacteria detected and 2.76 log_10_ CFU (data not shown; metadata original publication). By contrast, in the Ahmed DO mouse studies, mice were infected with the same target dose of M.tb within each experiment and progressors and controllers were established by the natural heterogeneity of the DO outbred mouse strain in inflammatory and protective responses following M.tb infection. Lung bacterial burden in controllers ranged between 3.78 and 5.65 log_10_ CFU (data not shown; metadata original publication). This may suggest that in this context, the signature scores are better able to differentiate between a clear variation in bacterial burden versus more complex immune determinants of disease progression at play in the DO mouse model in which the difference in lung bacterial burden between controllers and progressors is less resolved.

#### Resolution of signature score expression is lost at higher infection doses

**Fig 3:**
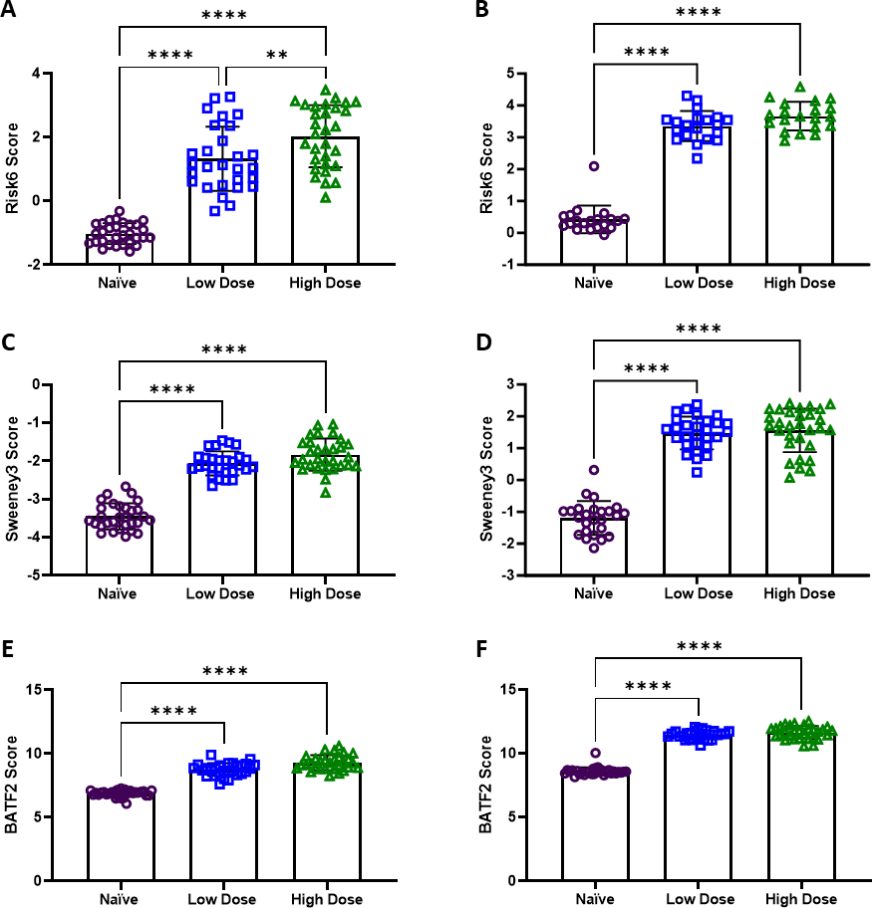
Risk signature scores assigned to naïve (purple circles), low dose (blue squares), or high dose (green triangles) C57BL/6 or C3HeB/FeJ mouse WB and lung samples. In Moreira-Teixeira et al. C57BL/6 or C3HeB/FeJ mice were infected with a low dose (100–450 CFU) or high dose (700–900 CFU) of M.tb and (**A, C, E**) WB (GSE137092) and (**B, D, F**) lung samples (GSE137093) were collected for gene expression analysis by RNA-seq. (**A, B**) Risk6, (**C, D**) Sweeney3, and (**E, F**) BATF2 scores were calculated for each animal, and cohorts were compared using one-way ANOVA with Tukey’s multiple comparison test correction. Significant comparisons are denoted by asterisks in the figure where ** = *P* ≤ 0.01, **** = *P* ≤ 0.0001.

In the final dose-related data set, we explored whether there were identifiable differences in risk score performance in infection conditions over 100 CFU. Moreira-Teixeira et al. (GSE137092 and GSE137093) infected C57BL/6 and C3HeB/FeJ mice with “low” (100–450 CFU) or “high” (700–900 CFU) doses of M.tb HN878 or H37Rv (43). WB and lung tissue were taken at 33–56 days post-infection. There were no significant differences between the strain of M.tb or mouse (data not shown), therefore downstream analyses with these groups were aggregated into naïve, low- and high-dose cohorts. There was a consistently significant difference in the WB and lung tissue scores between naïve, and animals infected with the high or low dose of M.tb (Fig. 3). The overlap in risk scores in WB and lung tissue observed between the two infection groups in this study indicate that Risk6, Sweeney3, and BATF2 are unable to differentiate between infection doses exceeding 100 CFU, resolving only on exposure to M.tb. Bacterial burden at the study end when WB and lung tissue samples were obtained exceeded 10^5^ log_10_ CFU in all animals (data not shown; Fig. S1 original publication). “High” dose groups did not exhibit consistently higher bacterial burdens at this time point compared with “low” dose groups.

### Kinetics

We next investigated whether the kinetics of infection would influence risk to active TB disease score performance. In humans, COR gene expression increases as an individual nears detectable disease progression, therefore we hypothesized we would observe similar kinetics in animal models as infections were established and bacterial burden increased over time. This reanalysis included 10 data sets meeting our inclusion/exclusion criteria (Table 4). This kinetic category of reanalysis spans two species (mouse—BALB/c, C57Bl/6, C3HeB/FeJ, 129S2; and NHP*—Macaca fascicularis*), three tissue types (WB, lung, and spleen), and five separate mycobacterial strains (*Mycobacterium bovis*, M.tb H37Rv, M.tb Erdman, and two Beijing isolates).

#### Kinetics of risk signature scores in WB and lung tissue

**Fig 4:**
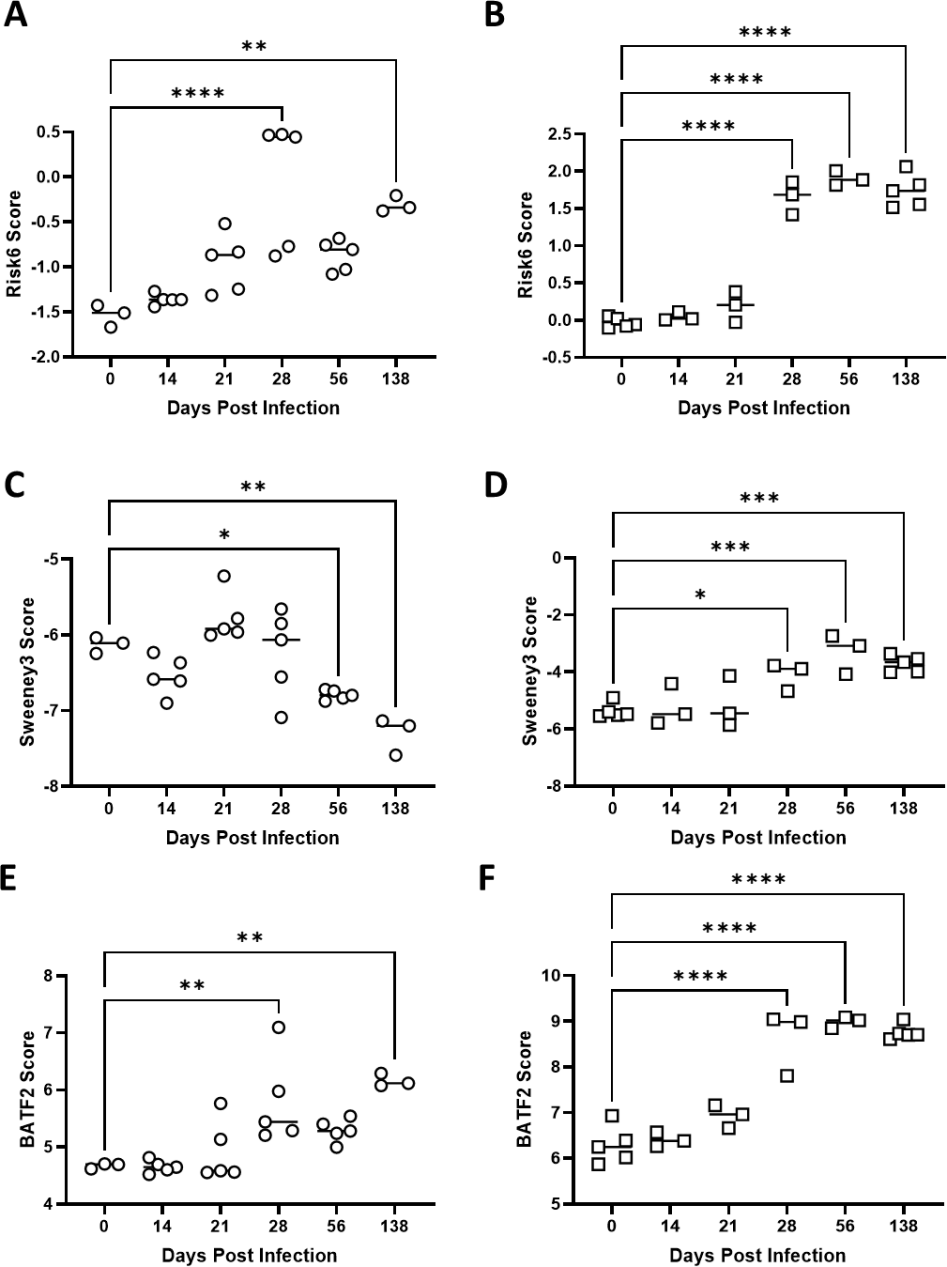
Risk signature scores derived from blood (open circles, **A, C, E**) or lung (open squares, **B, D, F**) tissue samples. In Moreira-Teixeira et al*.* (GSE140943 blood, left and GSE140944 lung, right), BALB/c mice were infected with a high dose (100–1,000 CFU) of M.tb H37Rv. Samples were evaluated for RNA analysis using the Illumina MouseWG-6 v2.0 expression beadchip and (**A, B**) Risk6, (**C, D**) Sweeney3, and (**E, F**) BATF2 scores were calculated for each animal. Scores were compared using one-way ANOVA with Dunnett’s multiple comparison test correction to tissue-matched day 0 scores. Significant comparisons are denoted by asterisks in the figure where * = *P* ≤ 0.05, ** = *P* ≤ 0.01, *** = *P* ≤ 0.001, **** = *P* ≤ 0.0001.

Moreira-Teixeira et al.’s study design includes BALB/c mice that were infected with 100–1,000 CFU M.tb H37Rv and contain both lung-derived (GSE140944) and blood-derived (GSE140943) data over time. RISK6, Sweeney3, and BATF2 scores were used to examine whether blood- and lung-derived signatures performed similarly or disparately over time (Fig. 4). Overall, lung-derived tissue scores (Fig. 4B, D, and F) showed a greater increase over time than matched signature blood-derived scores (Fig. 4A, C, and E). Sweeney3 scores derived from blood did not increase over time and performed poorly compared with other scores (Fig. 4C). For Risk6 and Batf2 signatures the lung- and blood-derived scores started to differentiate from baseline around 28 days post-infection (Fig. 4A, B, E, and F). These data suggest that both blood, the traditional human sampling strategy, and lung, could be viable tissue types for evaluating specific COR signatures in preclinical models of TB disease. Furthermore, given the lung is the primary site of infection and disease it is not surprising that the resolution of scores from this tissue may outperform blood-based signatures in some conditions.

#### Correlation between risk signature and days post-infection versus median days post-infection

The kinetic data sets available included study designs with low- or high-dose infection with M.tb, the lowest being 25 CFU, and contained widely variable ranges of time. To examine broad changes or influences of time since infection or infection dose, the Pearson correlation *R*^2^ value for each data set (correlation between risk signature and days post-infection) was calculated and graphed versus median days post-infection (Fig. S3; Table S3). We did not observe trends in correlations based on infection dose (Fig. S3). However, *R*^2^ values were higher in data sets with lower median days post-infection, particularly for Risk6 (Fig. S3A) and BATF2 (Fig. S3C) signatures.

#### Kinetics of risk signature scores in latent and active TB disease

In Gideon et al., cynomolgus macaques were infected with instillation of 25 CFU M.tb Erdman and followed for up to 180 days post-infection. WB was collected for RNA and analyzed by an Illumina HumanHT-12 V4.0 expression beadchip (49). NHPs were differentiated into active or latent TB groups at the termination of the study and retrospectively analyzed based on clinical diagnosis and intensity of pulmonary lesions with 18F-fludeoxyglucose and imaging. Using calculated risk signature scores, we observed that only the active disease group had a weak correlation (Spearman *r* = 0.5934, *P* = 0.0360) with time since infection, but no significant difference within the active or latent groups over time (Fig. S4). In this experiment, risk scores were not strongly associated with disease severity, nor were they able to differentiate active versus latent categorically by these endpoints.

A high initial infection dose appears to reduce the predictive ability of the risk signatures evaluated in this kinetics section. However, in a direct comparison of two sample types, risk signature scores derived from lung tissue exhibited better resolution across time than blood-based samples, and should be followed up in future work.

### Interventions

Most therapeutic treatment is designed to reduce *in vivo* bacterial burden either through direct bactericidal activity or host-mediated control. Therefore, we hypothesized that genetic interventions or treatment that resulted in expanding bacterial burden or dampened host immunity would subsequently result in higher COR scores. Conversely, we expected to observe decreasing COR gene expression through courses of successful drug treatment or vaccination. This reanalysis included 14 data sets meeting our inclusion/exclusion criteria (Table 5). This intervention category of reanalysis spans two species (mouse—C57Bl/6, Sp110, Sp140, A/Sn, I/St, ΔdblGata, DBA/2, BALB/c, B6D2F1, C3HeB/FeJ; and NHP*—Macaca mulatta*), three tissue types (blood, lung, and spleen), and involves four M.tb strains (Erdman, H37Rv, HN878, and CDC1551).

#### Influence of mechanistic (genetic, depletion, or metabolic) interventions on signature gene expression

**Fig 5:**
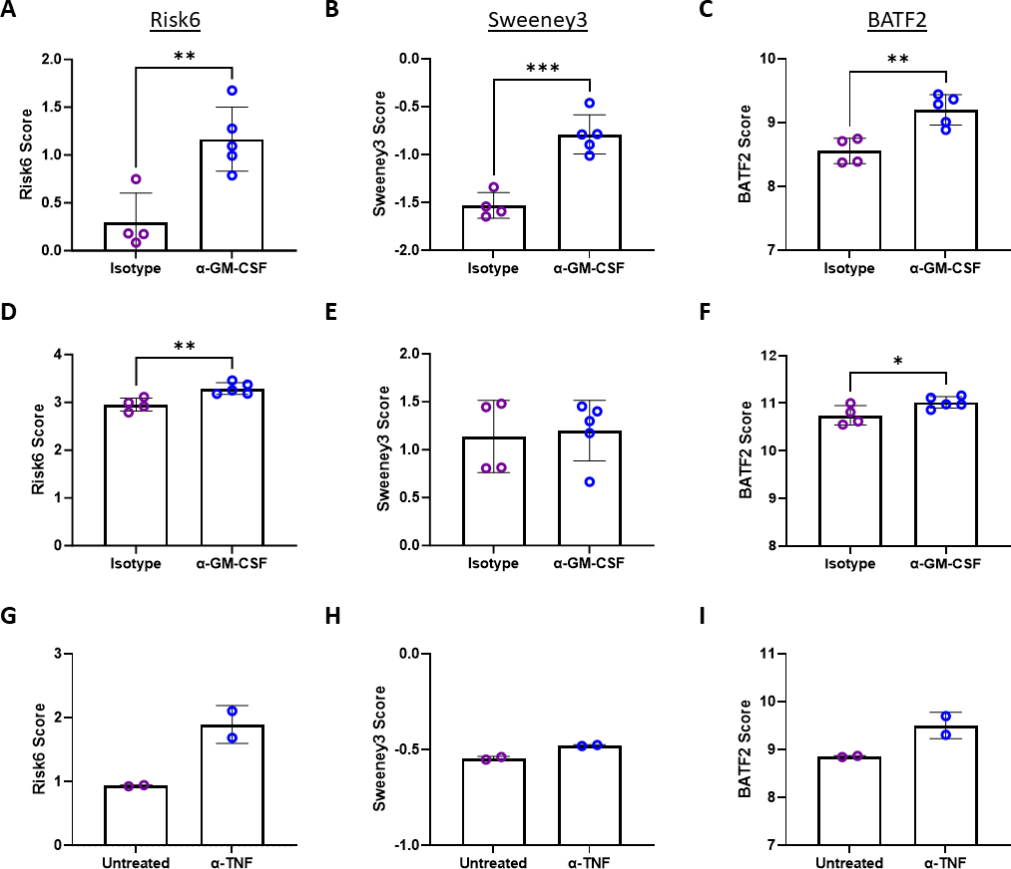
Risk signature scores increase with depletion of cytokines involved in M.tb control. (A–F) In Moreira-Teixeira et al*.* (GSE141207), C57BL/6 mice infected with 200 CFU M.tb HN878 and treated with either an isotype control (open purple circles) or α-GM-CSF depleting antibody (open blue circles) one day before infection and twice per week during the study. RNA from both (A–C) blood and (D–F) lung were collected for sequencing. (G–I) In Dutta et al*.* (GSE55183), C3HeB/FeJ mice were infected with ~1.0 OD_600_ M.tb H37Rv. After 6 weeks mice were left untreated (open purple circles) or given an α-TNF depleting antibody (open blue circles) twice per week for 4 weeks and lung tissue was collected for RNA analysis. (A, D, G) Risk6, (B, E, H) Sweeney3, and (C, F, I) BATF2 scores were calculated for each animal. In panels A–F groups were compared by *t*-test, but in panels G–I groups were not statistically compared due a group size of 2. Significant comparisons are denoted by asterisks in the figure where * = *P* ≤ 0.05, ** = *P* ≤ 0.01, and *** = *P* ≤ 0.001.

Deletion or depletion of key immune regulators is known to enhance preclinical model susceptibility to M.tb infection and allow higher bacterial burden. Interestingly, assessment of risk signature expression from lung samples derived from TB-susceptible I/St or TB-resistant A/Sn mice (67) in a study by Wilk et al. (GSE64167) did not reveal significant differences (data not shown). Similarly, in Bohrer et al. (GSE165871), mice lacking eosinophils, ΔdblGata, demonstrated equal Risk6, Sweeney3, and BATF2 scores from lung RNA samples compared to C57BL/6 mice after challenge with M.tb H37Rv (data not shown). The interest in connecting genetic loci to disease outcomes in mice has also revealed the super susceptibility to tuberculosis 1 (Sst1) locus. In the study by Ji et al. (GSE166114), we found trends (*n* = 2) of higher Risk6, Sweeney3, and BATF2 signature expression in RNA samples from M.tb Erdman challenged Sp140^−/−^ mice (lacking a repressor of type I IFN transcription) compared to C57BL/6 mice (data not shown). In the same study, the reverse trend was observed for Sp110^−/−^ mice (lacking NF-κβ modulating nuclear protein), where all signatures were lower than C57BL/6 mice (data not shown). Studies evaluating downstream immune effectors may further help identify the relationship between key immune regulators and risk signature expression during M.tb infection. For example, granulocyte-macrophage colony-stimulating factor (GM-CSF) has been shown to be an influential cytokine involved in the inhibition of intracellular M.tb growth (68). Moreira-Teixeira et al. (GSE141207) collected RNA from blood and lung tissue from C57BL/6 mice given either α-GM-CSF or isotype control, and subsequently infected with 200 CFU M.tb HN878. Infected cohorts, regardless of treatment group, harbored significantly higher risk signatures than naïve mice in both tissues (data not shown). When comparing risk signatures from infected animals we observed that all three signatures were more affected by α-GM-CSF treatment in blood samples (Fig. 5A through C) than in samples derived from lung tissue (Fig. 5D through F). In lung samples, there was no difference observed in the Sweeney3 signature (Fig. 5E), whereas all other conditions demonstrated a significant increase in score with α-GM-CSF treatment (Fig. 5). This difference between tissues and changes in risk signature expression may be partially due to penetrance of α-GM-CSF antibodies in solid tissue versus peripheral blood and the magnitude of changes that may subsequently occur. Tumor necrosis factor (TNF) is another important cytokine involved in the control of M.tb *in vivo* (69–71). In a study by Dutta et al. (GSE55183), they compared transcriptional differences from C3HeB/FeJ mice infected with M.tb H37Rv with or without α-TNF treatment. In lung samples derived from these experiments, *in vivo* depletion of TNF showed a trend of increasing COR signature expression. Statistics were not performed since the group size (*n* = 2) for this series was too small (Fig. 5G through I). These data suggest that in the same way reducing key cytokines can enhance susceptibility to M.tb in humans with immunosuppressive therapy, preclinical *in vivo* depletion of influential cytokines can increase TB disease risk signatures.

**Fig 6:**
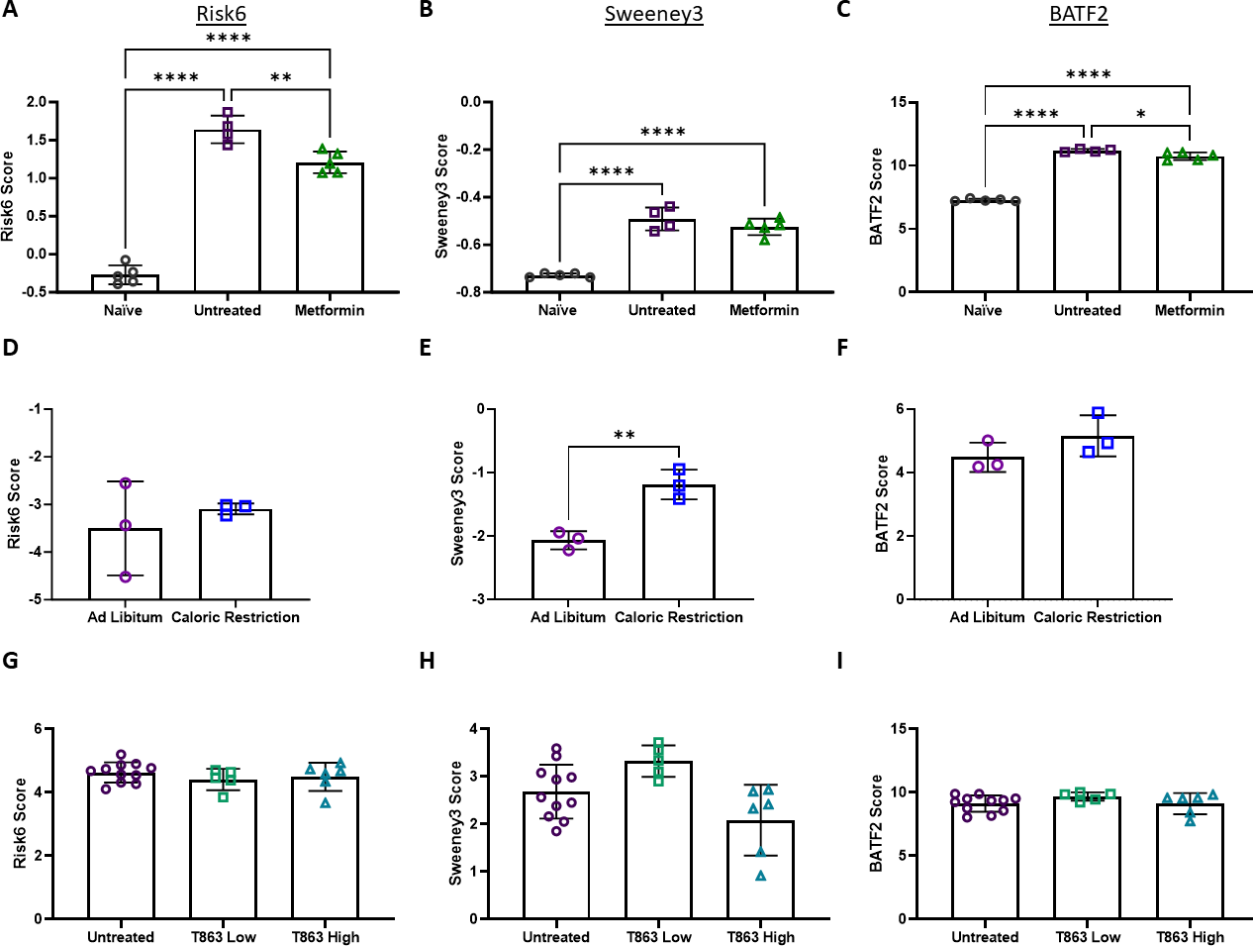
Risk signature scores are moderately influenced by metabolic interventions. (**A–C**) In Singal et al. (GSE57275), C57BL/6 mice were infected with 10^3^ CFU M.tb H37Rv for 7 days and left untreated (open purple squares) or treated with metformin (250 mg/kg, open green triangles) 6 days a week for 28 days. Lung tissue from these cohorts and naïve cohorts (open black circles) was collected for RNA analysis using Illumina MouseWG-6 v2.0 expression beadchip. (**D–F**) Matarese et al. (GSE127263) infected DBA/2 mice with 10^5^ CFU M.tb H37Rv by i.v. These animals were given *ad libitum* normal chow (open purple circles) or chow intended to result in caloric restriction (open blue squares). Diets were maintained for 40 days up until spleen tissue was collected for RNA-seq analysis. (**G–I**) In Gandotra et al. (GSE179437), C3HeB/FeJ mice were infected with 100 (low, open green squares) or 500 (high, open teal triangles) CFU M.tb Erdman. T863 was administered every 3 days from day 7 up until 28 via i.v. when lung tissues were collected and used for RNA-seq analysis along with untreated (open purple circles) animals. (**A, D, G**) Risk6, (**B, E, H**) Sweeney3, and (**C, F, I**) BATF2 scores were calculated for each animal, and cohorts were compared using an unpaired *t*-test where two groups are compared or an ordinary one-way ANOVA with Tukey’s multiple comparisons test where three or more groups are compared. Significant comparisons are denoted by asterisks in the figure where * = *P* ≤ 0.05, ** = *P* ≤ 0.01, **** = *P* ≤ 0.0001.

Given the significant roles of immunometabolism on host-pathogen outcomes we hypothesized that metabolic interferences may exacerbate M.tb infection and likewise influence genes in the POD risk signatures being evaluated. These data are particularly of interest given the recent success of the RATIONS clinical trial in Jharkhand India (reducing Activation of Tuberculosis by Improvement of Nutritional Status; Clinical Trial Registry of India: CTRI/2019/08/020490), where nutritional support to households with a member with active TB reduced incidence and subsequent infections significantly (72). This series of reanalyses includes RNA samples derived from spleen and lung tissues of mice infected with M.tb undergoing metformin treatment, caloric restriction, or treatment intended to lower triglycerides. Metformin given for 30 days reduced Risk6 and BATF2 signature scores compared with similarly infected but untreated mice (Fig. 6A and C) in data from Singal et al. (GSE57275). This trend was not observed for Sweeney3 (Fig. 6B). The reduction in signature scores in this data set was conservative, in line with the reductions in lung bacterial burden reported in the original publication. Conversely, in data from Matarese et al. (GSE127263), caloric restriction increased Sweeney3 scores when compared with chow given *ad libitum* in mice infected with M.tb, and not the other two scores tested, despite caloric restriction having a protective effect against bacterial burden *in vivo* as reported by the authors (Fig. 6D through F). Gandotra et al. (GSE179437) treated M.tb-infected C3HeB/FeJ mice from day 8 to 28 post-infection with compound T863, designed to inhibit triglyceride synthesizing enzyme DGAT1. In RNA derived from the lungs of these infected mice, treatment with T863 did not change signature score expression compared with untreated mice, even when segregated by low (100) or high (500 CFU) infection (Fig. 6G through I).

#### Impact of TB therapy on signature gene expression

**Fig 7:**
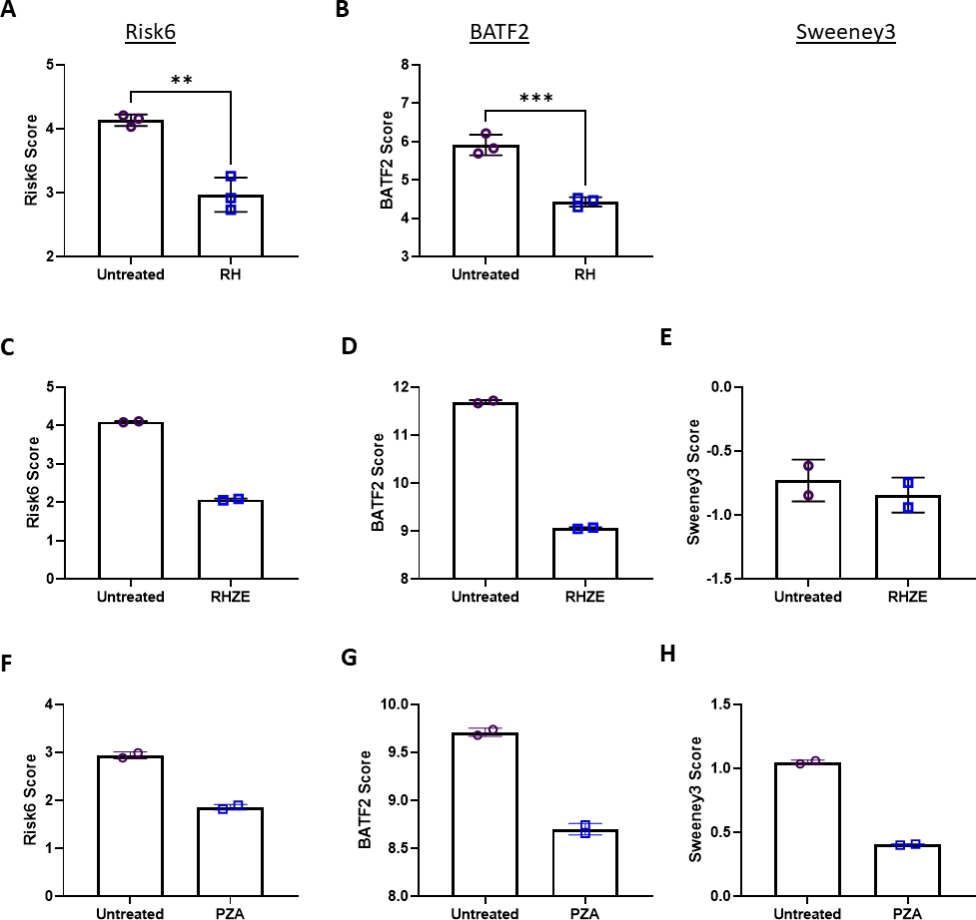
Risk signature scores from expression data derived from lungs of untreated (open purple circles) or drug-treated (open blue squares) mice. (**A and B**) In Rodrigues et al. (GSE44825), BALB/c mice infected with 10^5^ CFU M.tb H37Rv for 30 days and left untreated or treated for 30 days with rifampicin 20 mg/kg and isoniazid 25 mg/kg (RH) for 30 days. (**C–E**) Dutta et al. (GSE99625) infected BALB/c mice with 5 × 10^3^ CFU M.tb H37Rv and 6 weeks after infection treated with rifampicin 10 mg/kg + isoniazid 10 mg/kg + pyrazinamide 150 mg/kg + ethambutol 100 mg/kg (RHZE) orally for 4 weeks. (**F–H**) In a study by Manca et al*.* (GSE48027), B6D2F1 mice were infected with 250 CFU M.tb CDC1551 and then given pyrazinamide 150 mg/kg (PZA) for 35 days. (**A, C, F**) Risk6, (**B, D, G**) BATF2, and (**E, H**) Sweeney3 scores were calculated for each animal, and cohorts were compared using an unpaired *t*-test for (**A and B**) only where *n* = 3. Significant comparisons are denoted by asterisks in the figure where * = *P* ≤ 0.05, ** = *P* ≤ 0.01, *** = *P* ≤ 0.001. Scores were calculated for each animal, but not statistically evaluated when *n* = 2 per group (**C–H**).

This series of reanalyses included RNA samples derived from lung tissues of mice infected with M.tb and treated with various drug combinations. In highly effective and frontline antibiotic studies, decreasing Risk6 and BATF2 signatures correlate with treatment (Fig. 7). In the case of dual therapy with rifampicin (R) and isoniazid (H) for 30 days during the chronic phase of infection (>30 days post-infection), Rodrigues et al. (GSE44825) observed no detectable CFU after this treatment. A reanalysis of gene expression data from this study demonstrated that Risk6 and BATF2 scores are significantly decreased in the treated versus untreated cohort (Fig. 7A and B). Similarly, 4 weeks of RHZE (rifampicin, isoniazid, pyrazinamide, and ethambutol) treatment lowered both Risk6 and BATF2 scores but not Sweeney3 in a study by Dutta et al. (Fig. 7C through E). Pyrazinamide administered as a monotherapy for 35 days beginning at 28 days post-infection was found to reduce all three signatures compared with untreated animals (Fig. 7F through H).

**Fig 8:**
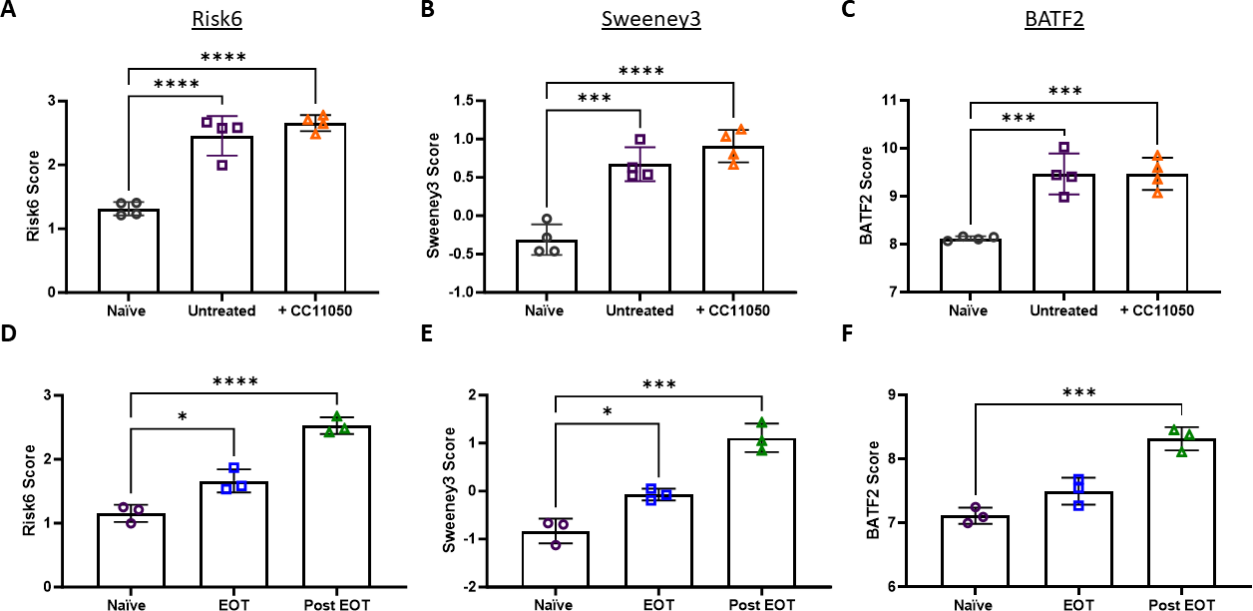
(**A–C**) Risk signature scores calculated from naïve (open black circles), infected but untreated (open purple squares), and infected and CC11050 treated (open orange triangles) B6D2F1 lung samples. In Subbian et al. (GSE83188), B6D2F1 mice were infected with 100–150 CFU M.tb CDC1551, and lung samples were collected for microarray analysis. (**D–F**) Risk signature scores were calculated from naïve (open purple circles), end-of-treatment (EOT, open blue squares), or post-treatment (Post-EOT, open green triangles) C57BL/6 lung samples. In Park et al*.* (GSE97835)*,* C57BL/6 mice were infected with 200–300 CFU M.tb Erdman, and lung samples were collected for microarray analysis. Samples were collected after treatment with a daily dose of INH: 26.5 ± 0.9 mg/kg; PZA and EMB: 132.6 ± 4.7 mg/kg at 4 weeks after treatment cessation. (**A, D**) Risk6, (**B, E**) Sweeney3, and (**C, F**) BATF2 scores were calculated for each animal, and cohorts were compared using one-way ANOVA with Tukey’s multiple comparison test correction. Significant comparisons are denoted by asterisks in the figure where * = *P* ≤ 0.05, *** = *P* ≤ 0.001, and **** = *P* ≤ 0.0001.

The above treatment regimens (RH, RHZE, and PZA) are known to reduce pulmonary bacterial burden and the risk signatures appear to track well with this trend. This holds when treatments are either ineffective or sub-optimal and result in “relapse-like” chronic infections. For example, Subbian et al. investigated whether compound CC-11050, a phosphodiesterase-4 inhibitor, would synergize with isoniazid (H) treatment (61). Importantly, CC-11050 treatment alone does not reduce bacterial burden compared with untreated mice in published literature and similarly, there is no difference in risk signature scores between these cohorts. However, there is an expected and significant increase in signature scores between naïve samples and both infection cohorts (Fig. 8A through C). We observe in Park et al. (GSE97835) that end-of-treatment (EOT) with HZE time points with persistent bacteria retain higher signature scores than naïve samples. After alleviation of treatment, wherein bacteria are expected to expand again, signature scores increase (post-EOT) (Fig. 8D through F).

#### Impact of vaccination on signature score expression

**Fig 9:**
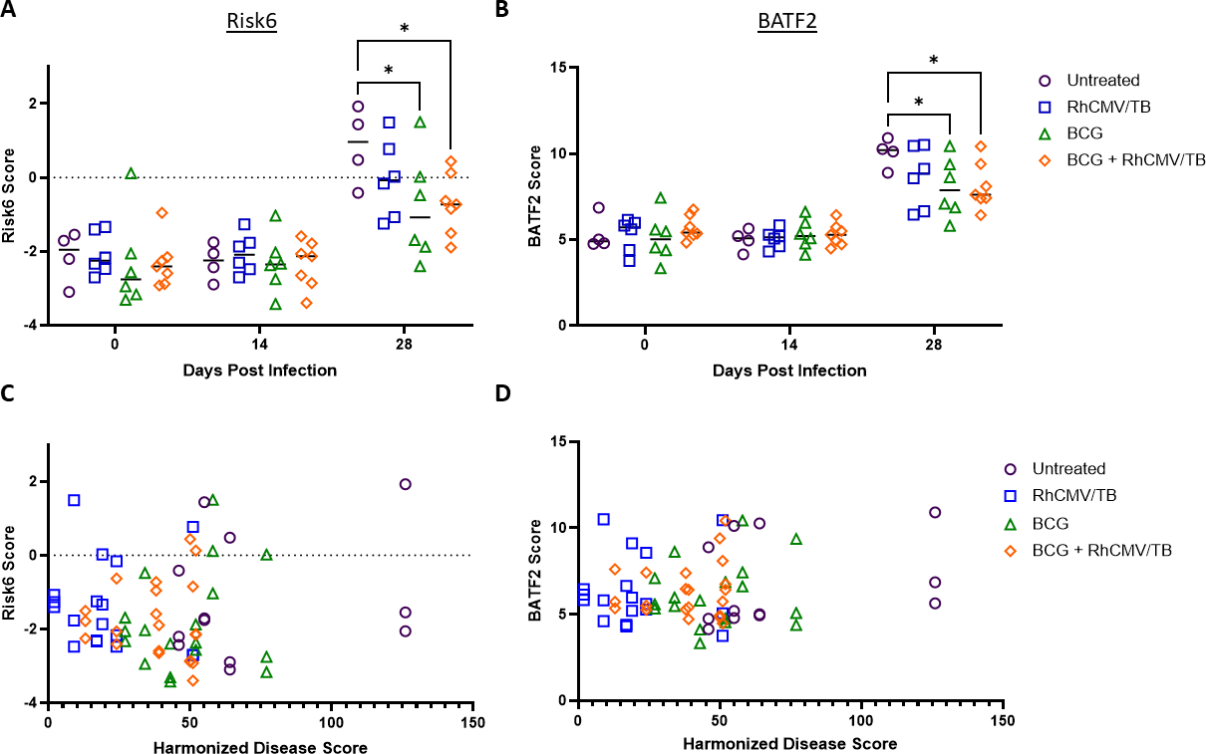
Risk signature scores derived from WB at 0, 14, or 28 days post-infection with M.tb Erdman. In Hansen et al*.* (GSE102440), cohorts of Rhesus macaques were left untreated (open purple circles), or vaccinated with BCG 68-1-9Ag (open blue squares), BCG (open green triangles), or 68-1-9Ag (open orange diamonds) and subsequently infected with 10–25 CFU M.tb Erdman and followed over time. (**A**) Risk6 and (**B**) BATF2 scores were calculated for each animal and cohorts were compared at each time point using a two-way ANOVA with Tukey’s multiple comparison test correction. Significant comparisons are denoted by asterisks in the figure where * = *P* ≤ 0.05. (**C**) Risk6 and (**D**) BATF2 scores were evaluated with harmonized disease scores for correlations using a Spearman-rank correlation test.

The final category of intervention-based data sets available included data from Hansen et al. (65) where rhesus macaques were vaccinated with BCG, RhCMV/TB candidate, or a prime-boost strategy of BCG with RhCMV/TB. RNA was extracted from WB in Paxgene tubes and analyzed by RNA-seq. After vaccination, NHPs were infected with 10–25 CFUs of M.tb Erdman. In all groups, the Risk6 scores increased significantly by 28 days post-infection compared with day 0 (day 0 vs 28 untreated *P* < 0.0001; RhCMV/TB *P* = 0.0006; BCG *P* = 0.0136; BCG + RhCMV/TB *P* = 0.0041). Additionally, when evaluating within day 28, groups with BCG regimens displayed significantly lower signatures compared with the untreated cohort at the same time point (Fig. 9A). Similarly, Batf2 scores were significantly higher for all of the groups by day 28 when compared with both day 0 and day 14 post-infection (day 0 vs 28 untreated *P* < 0.0001; RhCMV/TB *P* < 0.0001; BCG *P* = 0.0001; BCG + RhCMV/TB *P* = 0.0006). For Batf2 scores at day 28, groups with BCG regimens displayed significantly lower signatures compared with the untreated group (Fig. 9B). Each of the NHPs also had corresponding harmonized necropsy disease scores which were made up of the pathologic disease observed and recovered M.tb from tissue. These necropsy scores did not correlate significantly with the COR signature score (neither by group nor aggregated) (Fig. 9C and D).

These intervention-based data show an association between vaccine or drug treatment outcomes with COR gene signature scores, which may imply that these transcriptional scores could be used for early stage-gating of novel therapies against TB.

## DISCUSSION

In this reanalysis of public data sets, we aimed to determine whether human-derived risk signature scores are able to segregate naïve from infected animals and correlate with bacterial burden and/or disease burden endpoints in preclinical models of M.tb infection. A considerable number of predictive and diagnostic signatures of human TB disease have been published to date. This may, in part, be driven by the heterogeneity of immune responses and TB disease progression in different human clinical demographic groups (including unknown time since infection, when or how swiftly individuals will progress to active disease, etc.). Given animal studies have less intrinsic variability and known infection time and dose, we felt that analysis of selected TB biosignatures in published animal transcriptional data sets would be low-risk high-reward for the field. These data collectively support that Risk6, Sweeney3, and Batf2 risk signatures can be used as transcriptional endpoints in preclinical animals and that resolution is improved in specific conditions. The divergence between study designs, including dose and kinetic time points of sampling at first seemed a limitation of this reanalysis but has instead provided novel hypotheses about traditional endpoints that best correlate with signature scores. For example, lower infection doses invoke lower signature expression over time and can induce more variability across the group in line with trends in bacterial burden in these cohorts. In general, bacterial burden was found to more consistently correlate with higher signature gene expression compared with pulmonary immunopathology, imaging, or overt disease scoring. Additionally, early time points offered higher resolution than chronic time points captured, which is in line with lower initial bacterial deposition leading to a greater heterogeneity and resolution of scores. We hypothesize that with a higher initial infection dose, the COR signature kinetics are disrupted or reach an upper limit, making discrimination between groups more difficult. The mechanistic, drug, and vaccine-based intervention results support using COR signatures as endpoints to facilitate evaluation of therapeutic candidates in the preclinical pipeline. Risk signatures generated from WB and lung tissue could be used to support data from traditional CFU enumeration at study endpoints, with data generated more rapidly than CFU enumeration from plating organ homogenates. Furthermore, sampling of WB can be performed during an experiment providing a readout of efficacy prior to termination of the study.

One major limitation of these findings is that none of the studies in which the data sets were obtained for reanalysis were originally designed to address hypotheses related to COR score performance or identify confounding influences of study designs on COR scores. Follow-on studies designed and powered to do so are warranted given the proof-of-concept results presented here. For example, since we identified only two published data sets (GSE137092 and GSE137093) including more than one M.tb strain (H37Rv and HN878) we are unable to conclude whether M.tb strain or lineage impacts COR score performance or correlates with other clinical endpoints. We can hypothesize that this may be due to other factors having more influence and washing out strain-related effects. In the studies analyzed, no difference in signature score expression in whole blood or lung tissue samples was found (43). Highly virulent M.tb strains may promote enhanced COR gene expression given their relatively advanced kinetics of *in vivo* expansion. Sequencing of microbiologically confirmed human TB cases within clinical studies could also help address this question, however, sequencing is not routinely performed in many resource-limited areas at present. A second limitation of this analysis is that while some of the data sets analyzed included male and female animals, the metadata associated was not annotated with this information. Given the recent and exciting interest in sex-derived differences in TB disease and immune responses (73–75), evaluating whether gene signatures also diverge based on sex is worthwhile.

Future and active work in COR signatures includes the interrogation of the inherent mechanisms and networks of gene pathways to further understand the implications of score performance. Interestingly, Gupta et al. have previously used ingenuity pathway analysis to design a network of upstream gene or cytokine regulators of several COR gene signatures, including the three evaluated in this current re-analysis (32). In those analyses, a number of genes in Risk6, Sweeney3, and BATF2 feature prominently in *STAT1* and *IFNG* pathways, but not all. We hypothesize that the divergences are what differentiate score performance within sample sets as observed here. As more work is done to determine whether COR genes are directly tied to the mechanisms of disease progression or are merely an associated readout, having homologous signatures may accelerate the cross-pipeline validations and discoveries. Since these scores have shown promise in samples derived from *in vivo* preclinical models, originally excluded single-cell RNA-seq data could be leveraged to evaluate which cells contribute to signature gene expression across *in vitro* and *ex vivo* conditions, generating and validating novel hypotheses. The data evaluated here were exclusively derived from mouse or NHP-derived tissues. Several rabbit data sets were identified in our searches, but true orthologs of selected human gene sets of interest were difficult to confirm with confidence. A GP data set was also found in our searches but was insufficiently powered (*n* = 1/group). Intermediate models such as GPs and rabbits which display more similarities of human disease pathology compared with mice, but are less expensive and more readily available than NHPs, lack sufficient immunologic tools at present. However, once these needs are met, we expect that both GPs and rabbits will become valuable validation benchmarks for COR signatures as stage-gating endpoints. We predict that novel signatures will be identified that are high-performing but unique to specific species due to innate model differences or non-overlapping gene orthologs. These novel species-specific biomarkers are highly valuable for the preclinical TB vaccine pipeline and should be pursued, but may be limited in their inherent translation. These data demonstrate that human gene signature analysis can be applied to WB and lung tissue obtained from preclinical NHP and mouse models. In a number of data sets, scores were found to correlate with bacterial burden, indicating that human COR scores may provide a method to track infection progression within studies, and may represent a valuable tool to complement the evaluation of novel TB therapies.

## MATERIALS AND METHODS

### Study design

Using the GEO repository the following search terms were used: “mus tuberculosis AND Mus musculus” (69 total hits), “macaca tuberculosis” (5 total hits), “oryctolagus tuberculosis” (4 total hits), and “cavia tuberculosis” (1 hit). Within Pubmed, combinations of the following terms were used to search for data sets: tuberculosis, M.tb, biomarkers, RNA, risk scores, preclinical models, mouse, NHP, GPs, rabbit, challenge, microarray, sequencing, host gene expression, vaccine, or drug treatment. Data from cell lines, primary sorted cells, or single-cell RNA-seq were excluded. We required that included data be from primary reports and not review publications, that the data include at least one signature of interest, and groups contain at least two animals per group.

### Microarray data set pre-processing

All microarray data sets were processed in the R environment (v3.6.1). Normalized microarray data sets were downloaded using the getGEO function of the GEOquery package (76) and log_2_-transformed where required. Where data sets were incomplete or uploaded to GEO in an indiscernible format, raw files were downloaded and processed. For Agilent arrays (GSE21149), .txt files were downloaded, and background corrected (Normexp), quantile normalized, and filtered for expressed probes using limma (77). For Affymetrix arrays (GSE97835), .CEL files were downloaded, and background correction and normalization were performed using the rma function of the oligo package (78).

### RNA-seq data set pre-processing

Gene count tables were downloaded using the getGEO function of the GEOquery package. Where required, a normalized counts matrix was generated using DESeq2 (v1.28.1) (79) in the R environment (v3.6.1). GSE102440 was processed from raw fastq files since the original assembly used to map the reads was missing a gene required for signature generation. Sample processing from the download of .sra files to the generation of a summarized read count matrix was performed on a central shared Linex-based high-performance computing service at LSHTM. Sra.toolkit (SRA Toolkit Development Team, https://trace.ncbi.nlm.nih.gov/Traces/sra/sra.cgi?view=software) was used to download fastq files for BioProject PRJNA397748. STAR (v2.7.3a) (80) was used to generate a genome index and subsequently map the reads to the *Macaca mulatta* Mmul_10 assembly. Read counting was performed using the default settings of featureCounts (81) specifying a reversely stranded data set. Quality control steps were performed using FastQC (v0.11.9) (S. Andrews, FastQC: a quality control tool for high throughput sequence data, 2010, http://www.bioinformatics.babraham.ac.uk/projects/fastqc/) and CollectRnaSeqMetrics from the Picard Tools suite (Broad Institute, http://broadinstitute.github.io/picard/). A normalized counts matrix was generated as described above.

### Generation of signature scores

RISK6 (27) and Sweeney3 (35) signature scores, as well as BAFT2 (36) transcript levels, were generated from each data set as described previously using normalized log_2_-transformed mean fluorescence intensity values (microarray) or normalized read count values (RNA-seq).

### Statistics

COR signature scores were calculated for each animal and cohorts were compared across conditions using one-way analysis of variance (ANOVA) with Tukey’s or Dunnetts multiple comparison test correction where there were >3 groups or evaluations were over time. Student’s *t*-test was used for comparison of two groups at a single time point. Significant comparisons are denoted by asterisks in the figure where * = *P* ≤ 0.05, ** = *P* ≤ 0.01, *** = *P* ≤ 0.001, **** = *P* ≤ 0.0001. Data sets that included animal-matched transcriptional results with time, bacterial burden, or pathology endpoint results were evaluated for correlations with the Spearman rank-based correlation test, and r and *P* values were reported. Where too few data points were available to meet the requirements of a Spearman correlation, a Pearson correlation was instead used and *R*^2^ and *P* values are reported where appropriate.

## Data Availability

All data are available in the text or the supplemental material. Complete lists of accession numbers, first authors, and preclinical models for all data relating to the reanalysis performed in this paper are given in Tables 3 to 5.

## SUPPLEMENTAL MATERIAL

Supplemental material msphere.00864-24-s0001.docx Supplemental tables and figures.

ASM does not own the copyrights to Supplemental Material that may be linked to, or accessed through, an article. The authors have granted ASM a non-exclusive, world-wide license to publish the Supplemental Material files. Please contact the corresponding author directly for reuse.

## References

[1] World Health Organization. 2020. Global tuberculosis report 2020: executive summary.

[2] World Health Organization. 2022. Global TB report.

[3] World Health Organization. 2023. Global tuberculosis report.

[4] World Health Organization. 2021. Global tuberculosis report.

[5] Zaman K. 2010. Tuberculosis: a global health problem. J Health Popul Nutr 28:111–113. doi:10.3329/jhpn.v28i2.4879 20411672 PMC2980871

[6] Centers for Disease Control. 1992. Prevention and control of tuberculosis among homeless persons: recommendations of the advisory council for the elimination of tuberculosis. MMWR Recomm Rep 41:13–23. 1314323

[7] Joshi R, Reingold AL, Menzies D, Pai M. 2006. Tuberculosis among health-care workers in low- and middle-income countries: a systematic review. PLoS Med 3:e494. doi:10.1371/journal.pmed.0030494 17194191 PMC1716189

[8] Uden L, Barber E, Ford N, Cooke GS. 2017. Risk of tuberculosis infection and disease for health care workers: an updated meta-analysis. Open Forum Infect Dis 4:ofx137. doi:10.1093/ofid/ofx137 28875155 PMC5575844

[9] Dowdy DW, Grant AD, Dheda K, Nardell E, Fielding K, Moore DAJ. 2017. Designing and evaluating interventions to halt the transmission of tuberculosis. J Infect Dis 216:S654–S661. doi:10.1093/infdis/jix320 29112743 PMC5853231

[10] McIntosh AI, Jenkins HE, Horsburgh CR, Jones-López EC, Whalen CC, Gaeddert M, Marques-Rodrigues P, Ellner JJ, Dietze R, White LF. 2019. Partitioning the risk of tuberculosis transmission in household contact studies. PLoS One 14:e0223966. doi:10.1371/journal.pone.0223966 31639145 PMC6804987

[11] Behr MA, Kaufmann E, Duffin J, Edelstein PH, Ramakrishnan L. 2021. Latent tuberculosis: two centuries of confusion. Am J Respir Crit Care Med 204:142–148. doi:10.1164/rccm.202011-4239PP 33761302 PMC8650795

[12] Drain PK, Bajema KL, Dowdy D, Dheda K, Naidoo K, Schumacher SG, Ma S, Meermeier E, Lewinsohn DM, Sherman DR. 2018. Incipient and subclinical tuberculosis: a clinical review of early stages and progression of infection. Clin Microbiol Rev 31:e00021-18. doi:10.1128/CMR.00021-18 PMC6148193 30021818

[13] O’Garra A, Redford PS, McNab FW, Bloom CI, Wilkinson RJ, Berry MPR. 2013. The immune response in tuberculosis. Annu Rev Immunol 31:475–527. doi:10.1146/annurev-immunol-032712-095939 23516984

[14] Pai M, Behr MA, Dowdy D, Dheda K, Divangahi M, Boehme CC, Ginsberg A, Swaminathan S, Spigelman M, Getahun H, Menzies D, Raviglione M. 2016. Tuberculosis. Nat Rev Dis Primers 2:16076. doi:10.1038/nrdp.2016.76 27784885

[15] Vynnycky E, Fine PE. 1997. The natural history of tuberculosis: the implications of age-dependent risks of disease and the role of reinfection. Epidemiol Infect 119:183–201. doi:10.1017/s0950268897007917 9363017 PMC2808840

[16] Frascella B, Richards AS, Sossen B, Emery JC, Odone A, Law I, Onozaki I, Esmail H, Houben R. 2021. Subclinical tuberculosis disease—a review and analysis of prevalence surveys to inform definitions, burden, associations, and screening methodology. Clin Infect Dis 73:e830–e841. doi:10.1093/cid/ciaa1402 32936877 PMC8326537

[17] Esmail H, Macpherson L, Coussens AK, Houben RMGJ. 2022. Mind the gap - Managing tuberculosis across the disease spectrum. EBioMedicine 78:103928. doi:10.1016/j.ebiom.2022.103928 35339424 PMC9044004

[18] Trajman A, Steffen RE, Menzies D. 2013. Interferon-gamma release assays versus tuberculin skin testing for the diagnosis of latent tuberculosis infection: an overview of the evidence. Pulm Med 2013:601737. doi:10.1155/2013/601737 23476763 PMC3582085

[19] Hamada Y, Gupta RK, Quartagno M, Izzard A, Acuna-Villaorduna C, Altet N, Diel R, Dominguez J, Floyd S, Gupta A, et al.. 2023. Predictive performance of interferon-gamma release assays and the tuberculin skin test for incident tuberculosis: an individual participant data meta-analysis. EClinMed 56:101815. doi:10.1016/j.eclinm.2022.101815 PMC9829704 36636295

[20] Scriba TJ, Fiore-Gartland A, Penn-Nicholson A, Mulenga H, Kimbung Mbandi S, Borate B, Mendelsohn SC, Hadley K, Hikuam C, Kaskar M, et al.. 2021. Biomarker-guided tuberculosis preventive therapy (CORTIS): a randomised controlled trial. Lancet Infect Dis 21:354–365. doi:10.1016/S1473-3099(20)30914-2 33508224 PMC7907670

[21] Mulenga H, Musvosvi M, Mendelsohn SC, Penn-Nicholson A, Kimbung Mbandi S, Fiore-Gartland A, Tameris M, Mabwe S, Africa H, Bilek N, et al.. 2021. Longitudinal dynamics of a blood transcriptomic signature of tuberculosis. Am J Respir Crit Care Med 204:1463–1472. doi:10.1164/rccm.202103-0548OC 34520313 PMC8865716

[22] Bayaa R, Ndiaye MDB, Chedid C, Kokhreidze E, Tukvadze N, Banu S, Uddin MKM, Biswas S, Nasrin R, Ranaivomanana P, Raherinandrasana AH, Rakotonirina J, Rasolofo V, Delogu G, De Maio F, Goletti D, Endtz H, Ader F, Hamze M, Ismail MB, Pouzol S, Rakotosamimanana N, Hoffmann J, HINTT working group within the GABRIEL network. 2021. Multi-country evaluation of RISK6, a 6-gene blood transcriptomic signature, for tuberculosis diagnosis and treatment monitoring. Sci Rep 11:13646. doi:10.1038/s41598-021-93059-1 34211042 PMC8249600

[23] Sutherland JS, van der Spuy G, Gindeh A, Thuong NTT, Namuganga A, Owolabi O, Mayanja-Kizza H, Nsereko M, Thwaites G, Winter J, Dockrell HM, Scriba TJ, Geluk A, Corstjens P, Stanley K, Richardson T, Shaw JA, Smith B, Malherbe ST, Walzl G, TrENDx-TB Consortium. 2022. Diagnostic accuracy of the cepheid 3-gene host response fingerstick blood test in a prospective, multi-site study: interim results. Clin Infect Dis 74:2136–2141. doi:10.1093/cid/ciab839 34550342 PMC9258935

[24] Berry MPR, Graham CM, McNab FW, Xu Z, Bloch SAA, Oni T, Wilkinson KA, Banchereau R, Skinner J, Wilkinson RJ, Quinn C, Blankenship D, Dhawan R, Cush JJ, Mejias A, Ramilo O, Kon OM, Pascual V, Banchereau J, Chaussabel D, O’Garra A. 2010. An interferon-inducible neutrophil-driven blood transcriptional signature in human tuberculosis. Nature 466:973–977. doi:10.1038/nature09247 20725040 PMC3492754

[25] Walter ND, Miller MA, Vasquez J, Weiner M, Chapman A, Engle M, Higgins M, Quinones AM, Rosselli V, Canono E, Yoon C, Cattamanchi A, Davis JL, Phang T, Stearman RS, Datta G, Garcia BJ, Daley CL, Strong M, Kechris K, Fingerlin TE, Reves R, Geraci MW. 2016. Blood transcriptional biomarkers for active tuberculosis among patients in the United States: a case-control study with systematic cross-classifier evaluation. J Clin Microbiol 54:274–282. doi:10.1128/JCM.01990-15 26582831 PMC4733166

[26] Kaforou M, Wright VJ, Oni T, French N, Anderson ST, Bangani N, Banwell CM, Brent AJ, Crampin AC, Dockrell HM, Eley B, Heyderman RS, Hibberd ML, Kern F, Langford PR, Ling L, Mendelson M, Ottenhoff TH, Zgambo F, Wilkinson RJ, Coin LJ, Levin M. 2013. Detection of tuberculosis in HIV-infected and -uninfected African adults using whole blood RNA expression signatures: a case-control study. PLoS Med 10:e1001538. doi:10.1371/journal.pmed.1001538 24167453 PMC3805485

[27] Penn-Nicholson A, Mbandi SK, Thompson E, Mendelsohn SC, Suliman S, Chegou NN, Malherbe ST, Darboe F, Erasmus M, Hanekom WA, et al.. 2020. RISK6, a 6-gene transcriptomic signature of TB disease risk, diagnosis and treatment response. Sci Rep 10:8629. doi:10.1038/s41598-020-65043-8 32451443 PMC7248089

[28] Suliman S, Thompson EG, Sutherland J, Weiner J 3rd, Ota MOC, Shankar S, Penn-Nicholson A, Thiel B, Erasmus M, Maertzdorf J, et al.. 2018. Four-gene pan-African blood signature predicts progression to tuberculosis. Am J Respir Crit Care Med 197:1198–1208. doi:10.1164/rccm.201711-2340OC 29624071 PMC6019933

[29] Zak DE, Penn-Nicholson A, Scriba TJ, Thompson E, Suliman S, Amon LM, Mahomed H, Erasmus M, Whatney W, Hussey GD, et al.. 2016. A blood RNA signature for tuberculosis disease risk: a prospective cohort study. Lancet 387:2312–2322. doi:10.1016/S0140-6736(15)01316-1 27017310 PMC5392204

[30] MacLean E, Broger T, Yerlikaya S, Fernandez-Carballo BL, Pai M, Denkinger CM. 2019. A systematic review of biomarkers to detect active tuberculosis. Nat Microbiol 4:748–758. doi:10.1038/s41564-019-0380-2 30804546

[31] Kim CH, Choi G, Lee J. 2023. Host blood transcriptional signatures as candidate biomarkers for predicting progression to active tuberculosis. Tuberc Respir Dis (Seoul) 86:94–101. doi:10.4046/trd.2022.0152 36912017 PMC10073607

[32] Gupta RK, Turner CT, Venturini C, Esmail H, Rangaka MX, Copas A, Lipman M, Abubakar I, Noursadeghi M. 2020. Concise whole blood transcriptional signatures for incipient tuberculosis: a systematic review and patient-level pooled meta-analysis. Lancet Respir Med 8:395–406. doi:10.1016/S2213-2600(19)30282-6 31958400 PMC7113839

[33] Plumlee CR, Duffy FJ, Gern BH, Delahaye JL, Cohen SB, Stoltzfus CR, Rustad TR, Hansen SG, Axthelm MK, Picker LJ, Aitchison JD, Sherman DR, Ganusov VV, Gerner MY, Zak DE, Urdahl KB. 2021. Ultra-low dose aerosol infection of mice with Mycobacterium tuberculosis more closely models human tuberculosis. Cell Host Microbe 29:68–82. doi:10.1016/j.chom.2020.10.003 33142108 PMC7854984

[34] Mulenga H, Zauchenberger C-Z, Bunyasi EW, Mbandi SK, Mendelsohn SC, Kagina B, Penn-Nicholson A, Scriba TJ, Hatherill M. 2020. Performance of diagnostic and predictive host blood transcriptomic signatures for tuberculosis disease: a systematic review and meta-analysis. PLoS One 15:e0237574. doi:10.1371/journal.pone.0237574 32822359 PMC7442252

[35] Sweeney TE, Braviak L, Tato CM, Khatri P. 2016. Genome-wide expression for diagnosis of pulmonary tuberculosis: a multicohort analysis. Lancet Respir Med 4:213–224. doi:10.1016/S2213-2600(16)00048-5 26907218 PMC4838193

[36] Roe JK, Thomas N, Gil E, Best K, Tsaliki E, Morris-Jones S, Stafford S, Simpson N, Witt KD, Chain B, Miller RF, Martineau A, Noursadeghi M. 2016. Blood transcriptomic diagnosis of pulmonary and extrapulmonary tuberculosis. JCI Insight 1:e87238. doi:10.1172/jci.insight.87238 27734027 PMC5053151

[37] Warsinske H, Vashisht R, Khatri P. 2019. Host-response-based gene signatures for tuberculosis diagnosis: a systematic comparison of 16 signatures. PLoS Med 16:e1002786. doi:10.1371/journal.pmed.1002786 31013272 PMC6478271

[38] Hamada Y, Penn-Nicholson A, Krishnan S, Cirillo DM, Matteelli A, Wyss R, Denkinger CM, Rangaka MX, Ruhwald M, Schumacher SG. 2022. Are mRNA based transcriptomic signatures ready for diagnosing tuberculosis in the clinic? - A review of evidence and the technological landscape. EBioMedicine 82:104174. doi:10.1016/j.ebiom.2022.104174 35850011 PMC9294474

[39] Warsinske HC, Rao AM, Moreira FMF, Santos PCP, Liu AB, Scott M, Malherbe ST, Ronacher K, Walzl G, Winter J, Sweeney TE, Croda J, Andrews JR, Khatri P. 2018. Assessment of validity of a blood-based 3-gene signature score for progression and diagnosis of tuberculosis, disease severity, and treatment response. JAMA Netw Open 1:e183779. doi:10.1001/jamanetworkopen.2018.3779 30646264 PMC6324428

[40] Bloom CI, Graham CM, Berry MPR, Rozakeas F, Redford PS, Wang Y, Xu Z, Wilkinson KA, Wilkinson RJ, Kendrick Y, et al.. 2013. Transcriptional blood signatures distinguish pulmonary tuberculosis, pulmonary sarcoidosis, pneumonias and lung cancers. PLoS One 8:e70630. doi:10.1371/journal.pone.0070630 23940611 PMC3734176

[41] Ahmed M, Thirunavukkarasu S, Rosa BA, Thomas KA, Das S, Rangel-Moreno J, Lu L, Mehra S, Mbandi SK, Thackray LB, Diamond MS, Murphy KM, Means T, Martin J, Kaushal D, Scriba TJ, Mitreva M, Khader SA. 2020. Immune correlates of tuberculosis disease and risk translate across species. Sci Transl Med 12:eaay0233. doi:10.1126/scitranslmed.aay0233 31996462 PMC7354419

[42] Koyuncu D, Niazi MKK, Tavolara T, Abeijon C, Ginese ML, Liao Y, Mark C, Specht A, Gower AC, Restrepo BI, Gatti DM, Kramnik I, Gurcan M, Yener B, Beamer G. 2021. CXCL1: a new diagnostic biomarker for human tuberculosis discovered using Diversity Outbred mice. PLoS Pathog 17:e1009773. doi:10.1371/journal.ppat.1009773 34403447 PMC8423361

[43] Moreira-Teixeira L, Tabone O, Graham CM, Singhania A, Stavropoulos E, Redford PS, Chakravarty P, Priestnall SL, Suarez-Bonnet A, Herbert E, Mayer-Barber KD, Sher A, Fonseca KL, Sousa J, Cá B, Verma R, Haldar P, Saraiva M, O’Garra A. 2020. Mouse transcriptome reveals potential signatures of protection and pathogenesis in human tuberculosis. Nat Immunol 21:464–476. doi:10.1038/s41590-020-0610-z 32205882 PMC7116040

[44] Ault RC, Headley CA, Hare AE, Carruthers BJ, Mejias A, Turner J. 2020. Blood RNA signatures predict recent tuberculosis exposure in mice, macaques and humans. Sci Rep 10:16873. doi:10.1038/s41598-020-73942-z 33037303 PMC7547102

[45] Aranday Cortes E, Kaveh D, Nunez-Garcia J, Hogarth PJ, Vordermeier HM. 2010. Mycobacterium bovis-BCG vaccination induces specific pulmonary transcriptome biosignatures in mice. PLoS One 5:e11319. doi:10.1371/journal.pone.0011319 20596522 PMC2893133

[46] Domaszewska T, Scheuermann L, Hahnke K, Mollenkopf H, Dorhoi A, Kaufmann SHE, Weiner J 3rd. 2017. Concordant and discordant gene expression patterns in mouse strains identify best-fit animal model for human tuberculosis. Sci Rep 7:12094. doi:10.1038/s41598-017-11812-x 28935874 PMC5608750

[47] Gautam US, McGillivray A, Mehra S, Didier PJ, Midkiff CC, Kissee RS, Golden NA, Alvarez X, Niu T, Rengarajan J, Sherman DR, Kaushal D. 2015. DosS Is required for the complete virulence of Mycobacterium tuberculosis in mice with classical granulomatous lesions. Am J Respir Cell Mol Biol 52:708–716. doi:10.1165/rcmb.2014-0230OC 25322074 PMC4491129

[48] Kang DD, Lin Y, Moreno JR, Randall TD, Khader SA. 2011. Profiling early lung immune responses in the mouse model of tuberculosis. PLoS One 6:e16161. doi:10.1371/journal.pone.0016161 21249199 PMC3020951

[49] Gideon HP, Skinner JA, Baldwin N, Flynn JL, Lin PL. 2016. Early whole blood transcriptional signatures are associated with severity of lung inflammation in cynomolgus macaques with Mycobacterium tuberculosis infection. J Immunol 197:4817–4828. doi:10.4049/jimmunol.1601138 27837110 PMC5289749

[50] María Irene C-C, Juan Germán R-C, Gamaliel L-L, Dulce Adriana M-E, Estela Isabel B, Brenda Nohemí M-C, Payan Jorge B, Zyanya Lucía Z-B, Myriam BDV, Fernanda C-G, Adrian O-L, Martha Isabel M, Rogelio H-P. 2021. Profiling the immune response to Mycobacterium tuberculosis Beijing family infection: a perspective from the transcriptome. Virulence 12:1689–1704. doi:10.1080/21505594.2021.1936432 34228582 PMC8265813

[51] Naqvi KF, Huante MB, Saito TB, Endsley MA, Gelman BB, Endsley JJ. 2021. Novel role for macrophage galactose-type lectin-1 to regulate innate immunity against Mycobacterium tuberculosis. J Immunol 207:221–233. doi:10.4049/jimmunol.2001276 34183369 PMC8702441

[52] Shepelkova G, Pommerenke C, Alberts R, Geffers R, Evstifeev V, Apt A, Schughart K, Wilk E. 2013. Analysis of the lung transcriptome in Mycobacterium tuberculosis-infected mice reveals major differences in immune response pathways between TB-susceptible and resistant hosts. Tuberculosis (Edinb) 93:263–269. doi:10.1016/j.tube.2012.11.007 23276693

[53] Ji DX, Witt KC, Kotov DI, Margolis SR, Louie A, Chevée V, Chen KJ, Gaidt MM, Dhaliwal HS, Lee AY, Nishimura SL, Zamboni DS, Kramnik I, Portnoy DA, Darwin KH, Vance RE. 2021. Role of the transcriptional regulator SP140 in resistance to bacterial infections via repression of type I interferons. Elife 10:e67290. doi:10.7554/eLife.67290 34151776 PMC8248984

[54] Bohrer AC, Castro E, Hu Z, Queiroz ATL, Tocheny CE, Assmann M, Sakai S, Nelson C, Baker PJ, Ma H, et al.. 2021. Eosinophils are part of the granulocyte response in tuberculosis and promote host resistance in mice. J Exp Med 218:e20210469. doi:10.1084/jem.20210469 34347010 PMC8348215

[55] Moreira-Teixeira L, Stimpson PJ, Stavropoulos E, Hadebe S, Chakravarty P, Ioannou M, Aramburu IV, Herbert E, Priestnall SL, Suarez-Bonnet A, Sousa J, Fonseca KL, Wang Q, Vashakidze S, Rodríguez-Martínez P, Vilaplana C, Saraiva M, Papayannopoulos V, O’Garra A. 2020. Type I IFN exacerbates disease in tuberculosis-susceptible mice by inducing neutrophil-mediated lung inflammation and NETosis. Nat Commun 11:5566. doi:10.1038/s41467-020-19412-6 33149141 PMC7643080

[56] Dutta NK, Illei PB, Jain SK, Karakousis PC. 2014. Characterization of a novel necrotic granuloma model of latent tuberculosis infection and reactivation in mice. Am J Pathol 184:2045–2055. doi:10.1016/j.ajpath.2014.03.008 24815353 PMC4076462

[57] Palma C, La Rocca C, Gigantino V, Aquino G, Piccaro G, Di Silvestre D, Brambilla F, Rossi R, Bonacina F, Lepore MT, et al.. 2021. Caloric restriction promotes immunometabolic reprogramming leading to protection from tuberculosis. Cell Metab 33:300–318. doi:10.1016/j.cmet.2020.12.016 33421383

[58] Singhal A, Jie L, Kumar P, Hong GS, Leow M-S, Paleja B, Tsenova L, Kurepina N, Chen J, Zolezzi F, Kreiswirth B, Poidinger M, Chee C, Kaplan G, Wang YT, De Libero G. 2014. Metformin as adjunct antituberculosis therapy. Sci Transl Med 6:263ra159. doi:10.1126/scitranslmed.3009885 25411472

[59] Dawa S, Menon D, Arumugam P, Bhaskar AK, Mondal M, Rao V, Gandotra S. 2021. Inhibition of granuloma triglyceride synthesis imparts control of Mycobacterium tuberculosis through curtailed inflammatory responses. Front Immunol 12:722735. doi:10.3389/fimmu.2021.722735 34603294 PMC8479166

[60] Dutta NK, Bruiners N, Zimmerman MD, Tan S, Dartois V, Gennaro ML, Karakousis PC. 2020. Adjunctive host-directed therapy with statins improves tuberculosis-related outcomes in mice. J Infect Dis 221:1079–1087. doi:10.1093/infdis/jiz517 31605489 PMC7325721

[61] Subbian S, Koo M-S, Tsenova L, Khetani V, Zeldis JB, Fallows D, Kaplan G. 2016. Pharmacologic inhibition of host phosphodiesterase-4 improves isoniazid-mediated clearance of Mycobacterium tuberculosis. Front Immunol 7:238. doi:10.3389/fimmu.2016.00238 27379099 PMC4911353

[62] Manca C, Koo M-S, Peixoto B, Fallows D, Kaplan G, Subbian S. 2013. Host targeted activity of pyrazinamide in Mycobacterium tuberculosis infection. PLoS One 8:e74082. doi:10.1371/journal.pone.0074082 24015316 PMC3755974

[63] Rodrigues RF, Zárate-Bladés CR, Rios WM, Soares LS, Souza PRM, Brandão IT, Masson AP, Arnoldi FGC, Ramos SG, Letourneur F, Jacques S, Cagnard N, Chiocchia G, Silva CL. 2015. Synergy of chemotherapy and immunotherapy revealed by a genome-scale analysis of murine tuberculosis. J Antimicrob Chemother 70:1774–1783. doi:10.1093/jac/dkv023 25687643

[64] Park S, Baek S-H, Cho S-N, Jang Y-S, Kim A, Choi I-H. 2017. Unique chemokine profiles of lung tissues distinguish post-chemotherapeutic persistent and chronic tuberculosis in a mouse model. Front Cell Infect Microbiol 7:314. doi:10.3389/fcimb.2017.00314 28752079 PMC5508001

[65] Hansen SG, Zak DE, Xu G, Ford JC, Marshall EE, Malouli D, Gilbride RM, Hughes CM, Ventura AB, Ainslie E, et al.. 2018. Prevention of tuberculosis in rhesus macaques by a cytomegalovirus-based vaccine. Nat Med 24:130–143. doi:10.1038/nm.4473 29334373 PMC5909823

[66] Sharpe S, White A, Gleeson F, McIntyre A, Smyth D, Clark S, Sarfas C, Laddy D, Rayner E, Hall G, Williams A, Dennis M. 2016. Ultra low dose aerosol challenge with Mycobacterium tuberculosis leads to divergent outcomes in rhesus and cynomolgus macaques. Tuberculosis (Edinb) 96:1–12. doi:10.1016/j.tube.2015.10.004 26786648

[67] Nikonenko BV, Averbakh MM Jr, Lavebratt C, Schurr E, Apt AS. 2000. Comparative analysis of mycobacterial infections in susceptible I/St and resistant A/Sn inbred mice. Tuber Lung Dis 80:15–25. doi:10.1054/tuld.1999.0225 10897380

[68] Rothchild AC, Stowell B, Goyal G, Nunes-Alves C, Yang Q, Papavinasasundaram K, Sassetti CM, Dranoff G, Chen X, Lee J, Behar SM. 2017. Role of granulocyte-macrophage colony-stimulating factor production by T cells during Mycobacterium tuberculosis infection. mBio 8:e01514-17. doi:10.1128/mBio.01514-17 29066547 PMC5654932

[69] Olsen A, Chen Y, Ji Q, Zhu G, De Silva AD, Vilchèze C, Weisbrod T, Li W, Xu J, Larsen M, Zhang J, Porcelli SA, Jacobs WR, Chan J. 2016. Targeting Mycobacterium tuberculosis tumor necrosis factor alpha-downregulating genes for the development of antituberculous vaccines. mBio 7:e01023-15. doi:10.1128/mBio.01023-15 PMC4895118 27247233

[70] Allie N, Grivennikov SI, Keeton R, Hsu N-J, Bourigault M-L, Court N, Fremond C, Yeremeev V, Shebzukhov Y, Ryffel B, Nedospasov SA, Quesniaux VFJ, Jacobs M. 2013. Prominent role for T cell-derived tumour necrosis factor for sustained control of Mycobacterium tuberculosis infection. Sci Rep 3:1809. doi:10.1038/srep01809 23657146 PMC3648802

[71] Jacobs M, Togbe D, Fremond C, Samarina A, Allie N, Botha T, Carlos D, Parida SK, Grivennikov S, Nedospasov S, Monteiro A, Le Bert M, Quesniaux V, Ryffel B. 2007. Tumor necrosis factor is critical to control tuberculosis infection. Microbes Infect 9:623–628. doi:10.1016/j.micinf.2007.02.002 17409008

[72] Bhargava A, Bhargava M, Velayutham B, Thiruvengadam K, Watson B, Kulkarni B, Singh M, Dayal R, Pathak RR, Mitra A, Rade K, Sachdeva KS. 2021. The RATIONS (Reducing Activation of Tuberculosis by Improvement of Nutritional Status) study: a cluster randomised trial of nutritional support (food rations) to reduce TB incidence in household contacts of patients with microbiologically confirmed pulmonary tuberculosis in communities with a high prevalence of undernutrition, Jharkhand, India. BMJ Open 11:e047210. doi:10.1136/bmjopen-2020-047210 PMC8141431 34016663

[73] Dabitao D, Bishai WR. 2023. Sex and gender differences in tuberculosis pathogenesis and treatment outcomes. Curr Top Microbiol Immunol 441:139–183. doi:10.1007/978-3-031-35139-6_6 37695428

[74] Dibbern J, Eggers L, Schneider BE. 2017. Sex differences in the C57BL/6 model of Mycobacterium tuberculosis infection. Sci Rep 7:10957. doi:10.1038/s41598-017-11438-z 28887521 PMC5591305

[75] Gupta M, Srikrishna G, Klein SL, Bishai WR. 2022. Genetic and hormonal mechanisms underlying sex-specific immune responses in tuberculosis. Trends Immunol 43:640–656. doi:10.1016/j.it.2022.06.004 35842266 PMC9344469

[76] Davis S, Meltzer PS. 2007. GEOquery: a bridge between the Gene Expression Omnibus (GEO) and BioConductor. Bioinformatics 23:1846–1847. doi:10.1093/bioinformatics/btm254 17496320

[77] Ritchie ME, Phipson B, Wu D, Hu Y, Law CW, Shi W, Smyth GK. 2015. limma powers differential expression analyses for RNA-sequencing and microarray studies. Nucleic Acids Res 43:e47. doi:10.1093/nar/gkv007 25605792 PMC4402510

[78] Carvalho BS, Irizarry RA. 2010. A framework for oligonucleotide microarray preprocessing. Bioinformatics 26:2363–2367. doi:10.1093/bioinformatics/btq431 20688976 PMC2944196

[79] Love MI, Huber W, Anders S. 2014. Moderated estimation of fold change and dispersion for RNA-seq data with DESeq2. Genome Biol 15:550. doi:10.1186/s13059-014-0550-8 25516281 PMC4302049

[80] Dobin A, Davis CA, Schlesinger F, Drenkow J, Zaleski C, Jha S, Batut P, Chaisson M, Gingeras TR. 2013. STAR: ultrafast universal RNA-seq aligner. Bioinformatics 29:15–21. doi:10.1093/bioinformatics/bts635 23104886 PMC3530905

[81] Liao Y, Smyth GK, Shi W. 2014. featureCounts: an efficient general purpose program for assigning sequence reads to genomic features. Bioinformatics 30:923–930. doi:10.1093/bioinformatics/btt656 24227677

