# Supplementary material for: Backtranslation of human RNA biosignatures of tuberculosis disease risk into the preclinical pipeline is condition dependent
Source: mSphere. 2024 Dec 9;10(1):e00864-24. doi: 10.1128/msphere.00864-24 (PMC11774039; doi:10.1128/msphere.00864-24)
Supplement: Supplemental material — Supplemental tables and figures. [file msphere.00864-24-s0001.docx]

Backtranslation of human RNA biosignatures of tuberculosis disease risk into the preclinical pipeline is condition dependent

**Authors:** Hannah Painter^1†^, Sasha E. Larsen^2†^, Brittany D. Williams^2,3^, Hazem F. M. Abdelaal^2^, Susan L. Baldwin^2^, Helen A. Fletcher^1^, Andrew Fiore-Gartland^4^, Rhea N. Coler^2,3,5*^.

**Supplementary Material**

| **Supplemental Table 1.** Risk signature gene function**.** | | |
| --- | --- | --- |
| **Signature** | **Gene composition** | **Protein: function** |
| **RISK6** | GBP2 | Guanylate-binding protein 2: GTPase that is interferon-inducible. |
|  | FCGR1B | Putative high affinity immunoglobulin gamma Fc receptor IB: proposed binding to immunoglobulin Fc region, involved in humoral immune response. |
|  | SERPING1 | Plasma protease C1 inhibitor: complexes with complement components C1r or C1s to sterically inactivate protease activity. |
|  | TUBGCP6 | Gamma-tubulin complex component 6: required component for the complex to nucleate microtubules at the centrosome. |
|  | TRMT2A | tRNA (uracil-5-)-methyltransferase homolog A: methyltransferase of primarily cytosolic tRNAs catalyzing methylation of uridine. |
|  | SDR39U1 | Epimerase family protein SDR39U1: expected NADP-dependent oxidoreductase. |
| **Sweeney3** | GBP5 | Guanylate-binding protein 5: GTPase that is interferon-inducible and promotes NLRP3 inflammasome assembly. |
|  | DUSP3 | Dual specificity protein phosphatase 3: dephosphorylates and inactivates signaling kinases ERK1 and ERK2. |
|  | KLF2 | Krueppel-like factor 2: transcription factor involved in the endothelial inflammatory response. |
| **BATF2** | BATF2 | Basic leucine zipper transcriptional factor ATF-like 2: transcription factor involved in immune cell fate and differentiation including dendritic cells. |
| All protein functions from UniProt.org reviewed pages. | | |

| **Supplemental Table 2.** Data captured in searches but excluded based on *a priori* criteria | | | | | | |
| --- | --- | --- | --- | --- | --- | --- |
| **GEO** | **author** | **Sample** | **Species** | **Year deposited** | **Platform** | **Rationale for exclusion** |
| GSE73654 | Kaushal | BAL | *Macaca mulatta* | 2015 | Agilent-015421 TNPRC Macaca mulatta 4x44k Array | No genes present from scores |
| GSE49947 | Subbian | Lung | *Oryctolagus cuniculus* | 2013 | Agilent-020908 Oryctolagus cuniculus (Rabbit) Oligo Microarray | No genes present from scores |
| GSE39219 | Subbian | Lung | *Oryctolagus cuniculus* | 2012 | Agilent-020908 Oryctolagus cuniculus (Rabbit) Oligo Microarray | No genes present from scores |
| GSE74687 | Subbian | Lung | *Oryctolagus cuniculus* | 2015 | Agilent-020908 Oryctolagus cuniculus (Rabbit) Oligo Microarray | No genes present from scores |
| GSE54442 | Subbian | Lung | *Oryctolagus cuniculus* | 2014 | Agilent-020908 Oryctolagus cuniculus (Rabbit) Oligo Microarray | No genes present from scores |
| GSE32447 | Jain | Lung | *Cavia porcellus* | 2011 | Genotypic Technology designed Custom Cavia porcellus 4x44k | Not suitable for generating scores due to one animal per group |
| GSE43337 | Nouailles | Lung | *Mus musculus* | 2013 | Agilent-014868 Whole Mouse Genome Microarray 4x44K G4122F | Not suitable for generating scores - raw data not uploaded, wt vs KO - data had to be raw data or normalized counts to be used in our data reanalysis |
| GSE74282 | Gengenbacher | Lymph node | *Mus musculus* | 2015 | Agilent-028005 SurePrint G3 Mouse GE 8x60K Microarray | No infection, vaccination only |
| GSE126355 | Nemeth | Alveolar macrophages | *Mus musculus* | 2019 | RNA-seq | Sorted cells |
| GSE58810 | Subbian | Lung | *Mus musculus* | 2014 | Affymetrix Mouse Gene 1.0 ST Array | No control group |
| GSE33094 | Subbian | Lung | *Oryctolagus cuniculus* | 2011 | Agilent-020908 Oryctolagus cuniculus (Rabbit) Oligo Microarray | No genes present from scores |
| GSE27992 | Subbian | Lung | *Oryctolagus cuniculus* | 2011 | Agilent-020908 Oryctolagus cuniculus (Rabbit) Oligo Microarray | No genes present from scores |
| GSE176139 | Subbian | Lung | *Oryctolagus cuniculus* | 2021 | RNA-seq | No genes present from scores |
| GSE169202 | Matsuyama | Lung | *Mus musculus* | 2021 | RNA-seq | *M. avium* and not M.tb challenge |

**
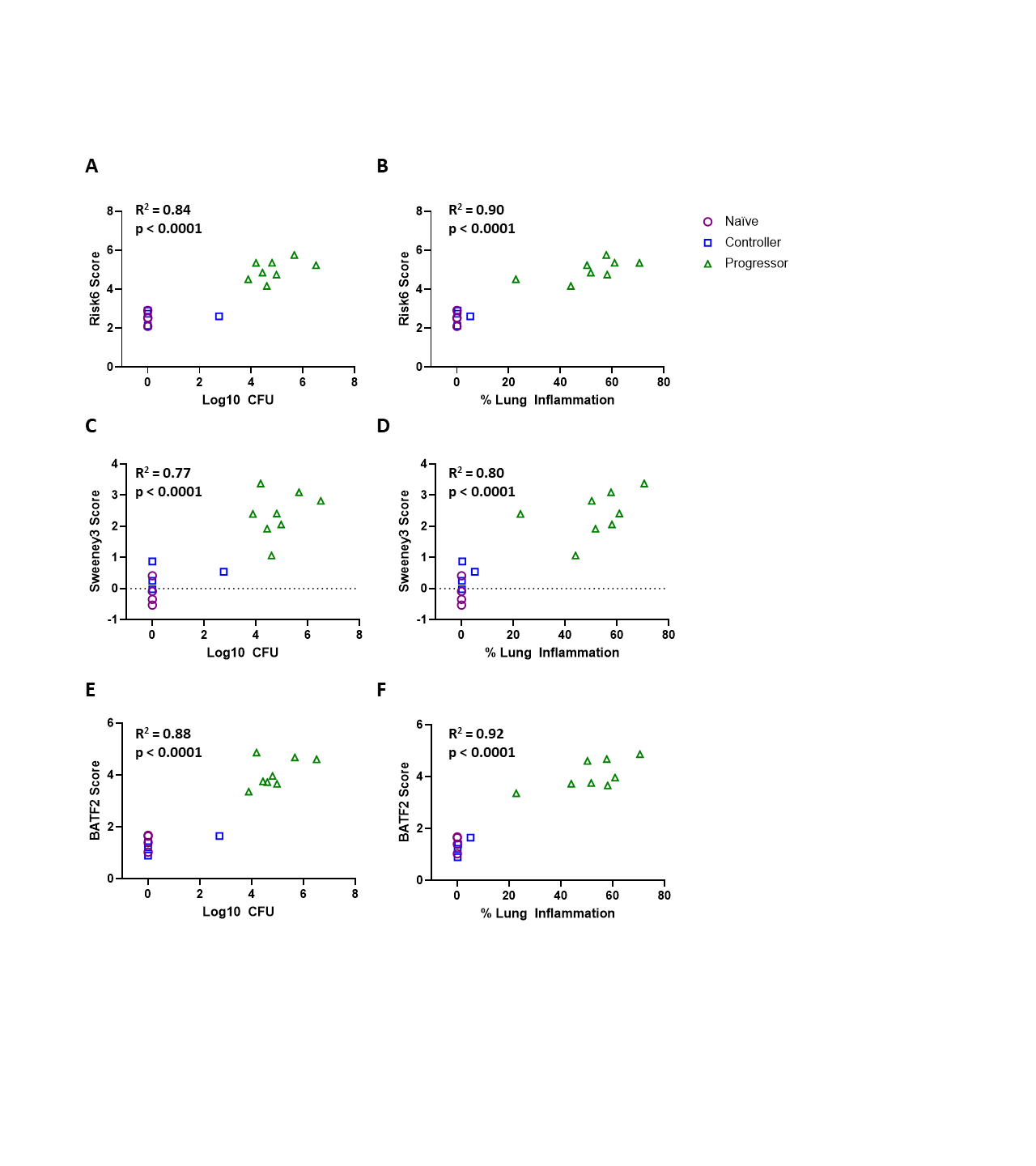
**

**Supplementary Figure 1.** Risk signatures correlate strongly with CFU and pulmonary inflammation in an NHP challenge model. In Ahmed *et al,* Indian rhesus macaques were left unchallenged (naïve, open purple circles), challenged with either 10 CFU and sampled at 5-8 weeks post infection (controllers, open blue square), or 100 CFU M.tb CDC1551 and samples collected 22-24 weeks post infection (progressors, open green triangle) for RNA sequencing. **A, B**) Risk6, **C, D**) Sweeney3 and **E, F**) BATF2 scores were calculated for each animal and compared by Pearson correlation with **A, C, E**) log10 CFU or **B, D, F**) percent lung inflammation. R^2^ and p values shown in the figure.

**
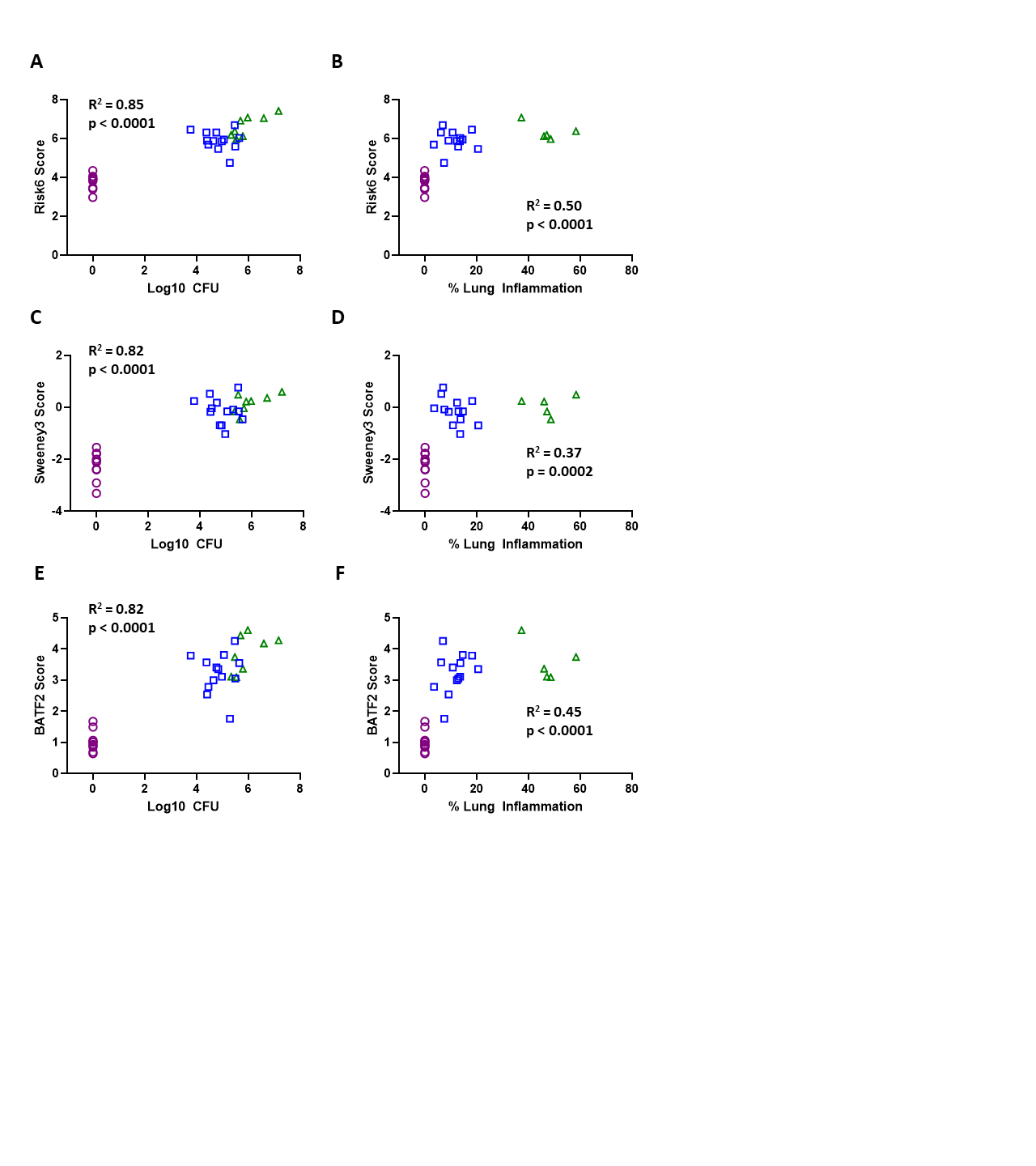
**

**Supplementary Figure 2.** Risk signatures correlate with CFU in a DO mouse model. In Ahmed *et al,* DO mice challenged with 100 CFU M.tb HN878 were assigned progressors or controllers based on “TB severity score”. Risk6, Sweeney3 and BATF2 scores were calculated for each animal and cohorts of naïve (purple), controllers (pink) or progressors (teal) were compared. **A, B**) Risk6, **C, D**) Sweeney3 and **E, F**) BATF2 scores were calculated for each animal and compared by Pearson correlation with **A, C, E**) log10 CFU or **B, D, F**) percent lung inflammation. R^2^ and p values shown in the figure.

**
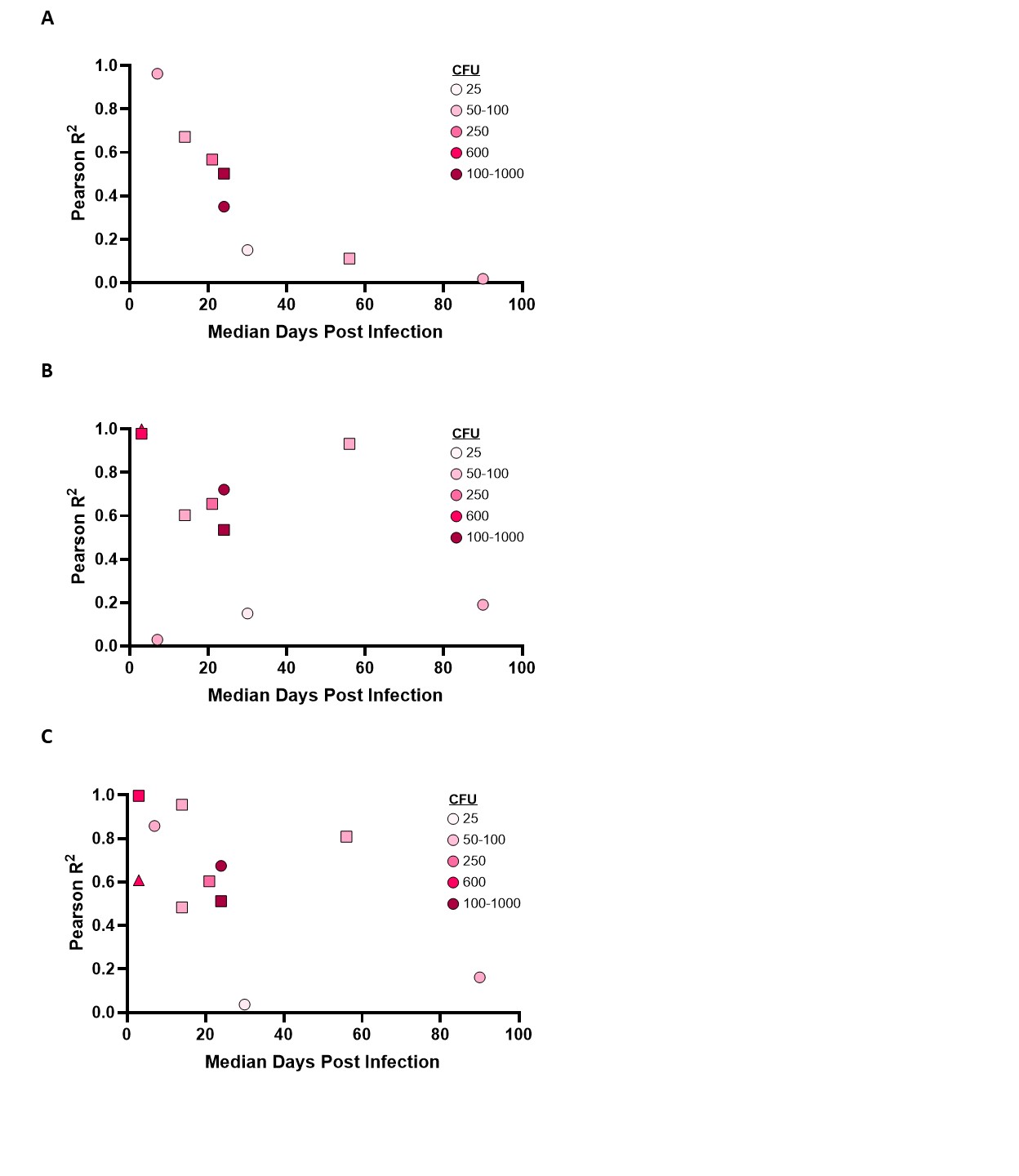
**

**Supplemental Figure 3.** Pearson correlation R^2^ of risk signature scores compared with time since infection. **A**) Risk6, **B**) Sweeney3 and **C**) BATF2 scores were calculated for each animal and a Pearson correlation was run evaluating the signature score versus time since challenge. Challenge dose is denoted by the color intensity of the shape (CFU 25 low to 100-1000 high). Samples derived from blood are denoted as circles, samples from lung are denoted as squares and those derived from spleen are triangles. Accompanying p values are listed in Supplemental Table 3.

| **Supplemental Table 3**. Pearson correlation R^2^ and p value by signature. | | | | | | | |
| --- | --- | --- | --- | --- | --- | --- | --- |
| **Dataset** | **Median days post infection** | **Risk6** | | **Sweeney3** | | **BATF2** | |
|  |  | R^2^ | p | R^2^ | p | R^2^ | p |
| GSE124688 | 90 | 0.017 | 0.8301 | 0.190 | 0.4621 | 0.161 | 0.5018 |
| GSE21149 Lung | 3 | x | x | 0.978 | 0.0937 | 0.995 | **0.0407** |
| GSE21149 Spleen | 3 | x | x | 0.999 | **0.015** | 0.608 | 0.4302 |
| GSE140944 | 24 | 0.503 | 0.1145 | 0.535 | 0.0982 | 0.512 | 0.1097 |
| GSE140943 | 24 | 0.350 | 0.2156 | 0.721 | **0.0324** | 0.674 | **0.045** |
| GSE89389 | 7 | 0.962 | **0.0031** | 0.0292 | 0.7832 | 0.857 | **0.0239** |
| GSE64045 | 56 | 0.111 | 0.7831 | 0.932 | 0.1674 | 0.808 | **0.288** |
| GSE23014 | 14 | 0.672 | 0.1803 | x | x | 0.483 | 0.3044 |
| GSE84152 | 30 | 0.150 | 0.1898 | 0.150 | 0.1898 | 0.0372 | 0.5277 |
| GSE169541 | 21 | 0.567 | 0.4572 | 0.655 | 0.3993 | 0.603 | 0.4334 |
| GSE168486 | 14 | x | x | 0.603 | 0.4338 | 0.955 | 0.1352 |
| x = genes not present for analysis in dataset | | | | | | | |


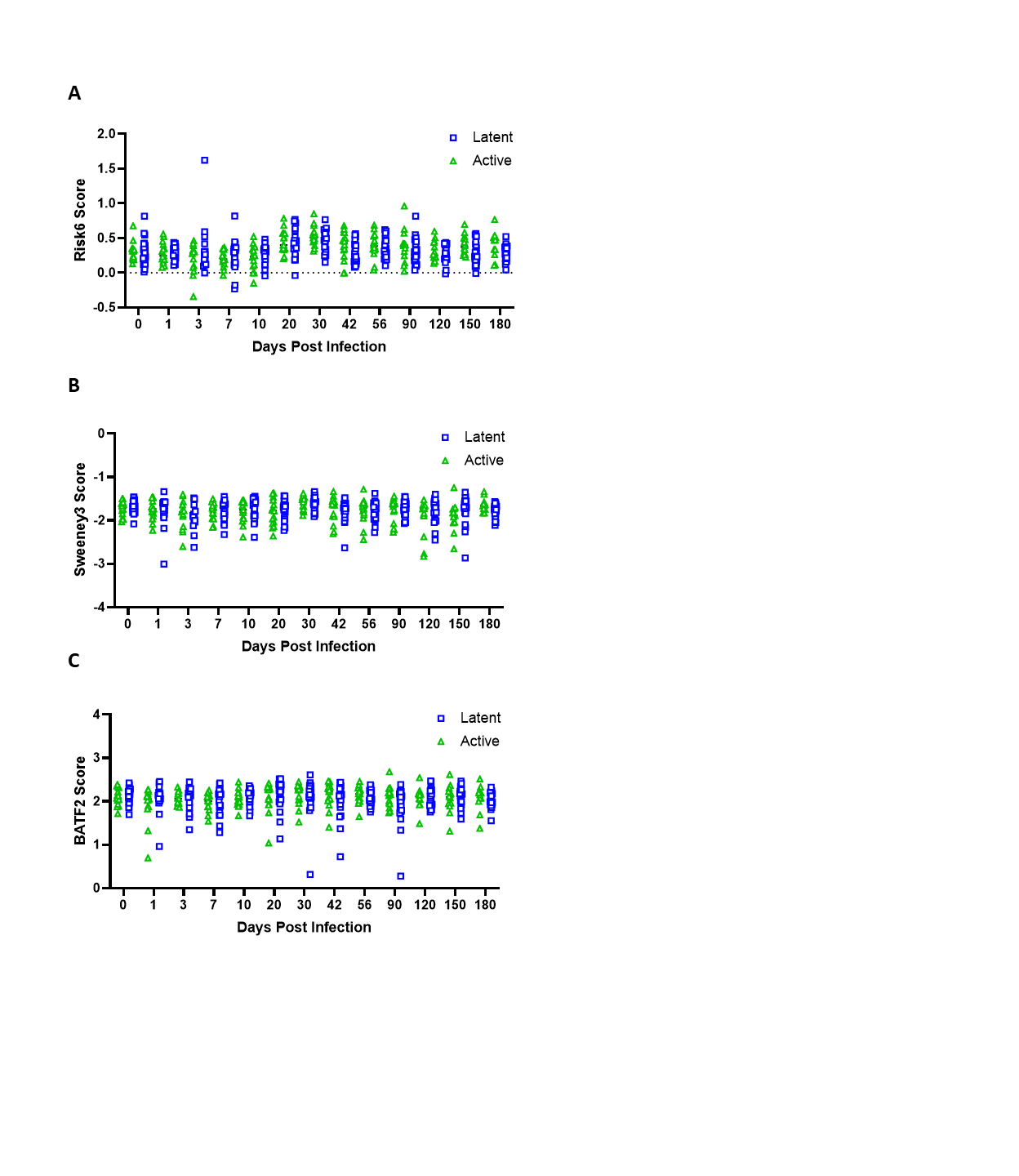


**Supplemental Figure 4.** Risk signature scores derived from WB samples from Cynomolgus macaques with either latent (open blue squares) or active TB disease (open green triangles). In Gideon *et al* (GSE84152) animals were infected with an instillation of 25 CFU M.tb Erdman and samples were taken from pre-infection through day 180 post-infection. Gene expression analysis was performed by Illumina HumanHT-12 V4.0 expression beadchip. **A**) Risk6, **B**) Sweeney3 and **C**) BATF2 scores were calculated for each animal and separate comparisons of active and latent cohorts were performed using one-way ANOVA with Tukey’s multiple comparison test correction.
